# Disruption of Treg Homeostasis in Rheumatoid Arthritis via Ferroptosis‐Mediated ETC Collapse and TXK‐STAT3/PLCγ1 Activation

**DOI:** 10.1002/advs.202520519

**Published:** 2026-05-19

**Authors:** Jingrong Chen, Xiao Guan, Kexu Xiong, Xiaoyi Shi, Luyao Wu, Chuanxin Su, Jun Zhao, Rongzhen Liang, Huimin Wang, Junlong Dang, Ye Chen, Yiding Xiong, Chi Zhou, Futao Zhao, Zhenhang Zhu, Xiaoli Fan, Li Zhou, Nancy Olsen, Yutong Jiang, Song Guo Zheng

**Affiliations:** ^1^ Division of Rheumatology Department of Medicine Songjiang Research Institute Songjiang Hospital Affiliated to Shanghai Jiao Tong University School of Medicine Shanghai China; ^2^ Department of Immunology School of Cell and Gene Therapy Songjiang Research Institute Songjiang Hospital Affiliated to Shanghai Jiao Tong University School of Medicine Shanghai China; ^3^ The State Key Laboratory of Innovative Immunotherapy At the Shanghai Jiao Tong University School of Medicine Shanghai China; ^4^ Shenzhen Key Laboratory of Viral Oncology The Clinical Innovation & Research Center (CIRC) Shenzhen Hospital Southern Medical University Shenzhen China; ^5^ Shenzhen Qianhai Shekou Free Trade Zone Hospital Shenzhen China; ^6^ Department of Organ Transplantation The Affiliated Guangdong Second Provincial General Hospital of Jinan University Guangzhou China; ^7^ Department of Cardiology Songjiang Research Institute Songjiang Hospital Affiliated to Shanghai Jiao Tong University School of Medicine Shanghai China; ^8^ Department of Rheumatology Ninth People's Hospital Shanghai Jiao Tong University School of Medicine Shanghai China; ^9^ Division of Oncology Department of Medicine Songjiang Hospital Affiliated to Shanghai Jiao Tong University School of Medicine Shanghai China; ^10^ Division of Rheumatology Department of Internal Medicine Penn State Hershey Medical Center Hershey Pennsylvania USA; ^11^ Division of Rheumatology Department of Internal Medicine The Third Affiliated Hospital of Sun Yat‐Sen University Guangzhou China

**Keywords:** autoimmunity, electron transport chain, ferroptosis, OXPHOS, regulatory T cell, rheumatoid arthritis

## Abstract

In rheumatoid arthritis (RA), regulatory T cells (Tregs) within the synovium present a paradox: they are numerically enriched yet functionally impaired, leading to a loss of immune tolerance. Here, we report that synovial iron overload establishes a ferroptosis‐permissive microenvironment that disrupts Treg homeostasis. Exposure to RA synovial fluid induced ferroptosis, autoimmune Treg‐associated metabolic shifts through lipid peroxide‐driven mitochondrial dysfunction, characterized by electron transport chain (ETC) collapse and impaired oxidative phosphorylation. Mechanistically, metabolic disturbance by ferroptotic stress or complex III blockade triggered TXK kinase upregulation, which is required for the phosphorylation of STAT3 (Tyr705) and PLCγ1 (Tyr783), activating a proinflammatory transcriptional program that destabilized Treg identity and promoted Th17‐like conversion. Crucially, this pathogenic reprogramming was reversed through iron chelation or TXK inhibition in vitro and in vivo. Our findings unveil a ferroptosis‐ETC‐TXK/STAT3 axis as a core mechanism of synovial Treg failure. Targeting synovial iron homeostasis or inhibiting TXK signaling thus represents a promising therapeutic strategy to restore immune tolerance in RA by rescuing Treg functionality.

## Graphical abstract

In rheumatoid arthritis, synovial Tregs accumulate but are functionally impaired due to iron overload‐induced ferroptosis. This triggers mitochondrial dysfunction and TXK tyrosine kinase‐mediated signaling, leading to Treg destabilization and inflammation. Targeting iron homeostasis or TXK restores Treg function, suggesting new therapeutic strategies.

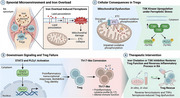

## 1 Introduction

Regulatory T cells (Tregs) play a pivotal role in maintaining peripheral tolerance and restraining autoimmune responses [1], yet paradoxically exhibit synovial‐specific functional impairment despite increased joint infiltration in rheumatoid arthritis (RA). Recent studies indicate that Tregs from the periphery of RA patients exhibit frequencies and in vitro suppressive capacities comparable to healthy controls [2]. Conversely, multiple investigations have demonstrated elevated Treg accumulation within the inflamed synovium [2, 3, 4]. This microenvironment‐specific functional attenuation represents a fundamental paradox in RA pathogenesis: why do synovial Tregs lose regulatory capacity while maintaining peripheral functionality? Current consensus confirms diminished Treg activity within RA joints [5, 6], highlighting the critical need to elucidate microenvironmental drivers of this impairment for developing targeted tolerance restoration strategies.

Notably, the RA‐inflamed joint exhibits distinct iron metabolic perturbations, as evidenced by magnetic resonance imaging and histology [7]. This iron accumulation, alongside elevated reactive oxygen species (ROS), is implicated in joint inflammation and bone destruction [7, 8]. Ferroptosis is mechanistically defined by glutathione depletion, glutathione peroxidase GPX4 inactivation, and lethal accumulation of iron‐catalyzed lipid peroxides [9, 10]. Recent studies suggest its broader pathophysiological relevance extends beyond stromal cells to immune dysregulation in autoimmunity [11, 12]. Dysregulated synovial iron metabolism may critically compromise the function of resident immune cells, including Tregs. While iron chelation strategies show therapeutic promise in models [13, 14], the precise mechanistic relationship between synovial iron overload, ferroptotic stress, and Treg impairment remains unexplored. Critically, Tregs exhibit metabolic vulnerabilities distinct from effector T cells, relying predominantly on mitochondrial respiration rather than glycolysis. This gap is particularly significant given the critical reliance of Tregs on mitochondrial oxidative phosphorylation (OXPHOS) for their functional integrity. We therefore hypothesize that synovial iron overload establishes a ferroptosis‐permissive niche that specifically targets Treg mitochondrial integrity, thereby disrupting their regulatory function.

Our data strongly suggest that synovial iron overload and the resultant ferroptotic stress constitute a key microenvironmental driver of Treg dysfunction in RA. This study aims to elucidate the causal chain linking synovial iron overload to metabolic dysregulation and functional impairment in Tregs. We specifically investigate how ferroptosis‐induced electron transport chain (ETC) damage disrupts Treg metabolism and how subsequent signaling cascades. Our findings establish TXK kinase as a novel metabolic stress sensor that translates ETC damage into STAT3/PLCγ1‐mediated Treg reprogramming—providing a mechanistic resolution to the synovial Treg paradox and revealing new targets for tolerance restoration in RA.

## 2 Results

### 2.1 Human Synovial Tregs Exhibit Heightened Ferroptosis Susceptibility in RA

**FIGURE 1:**
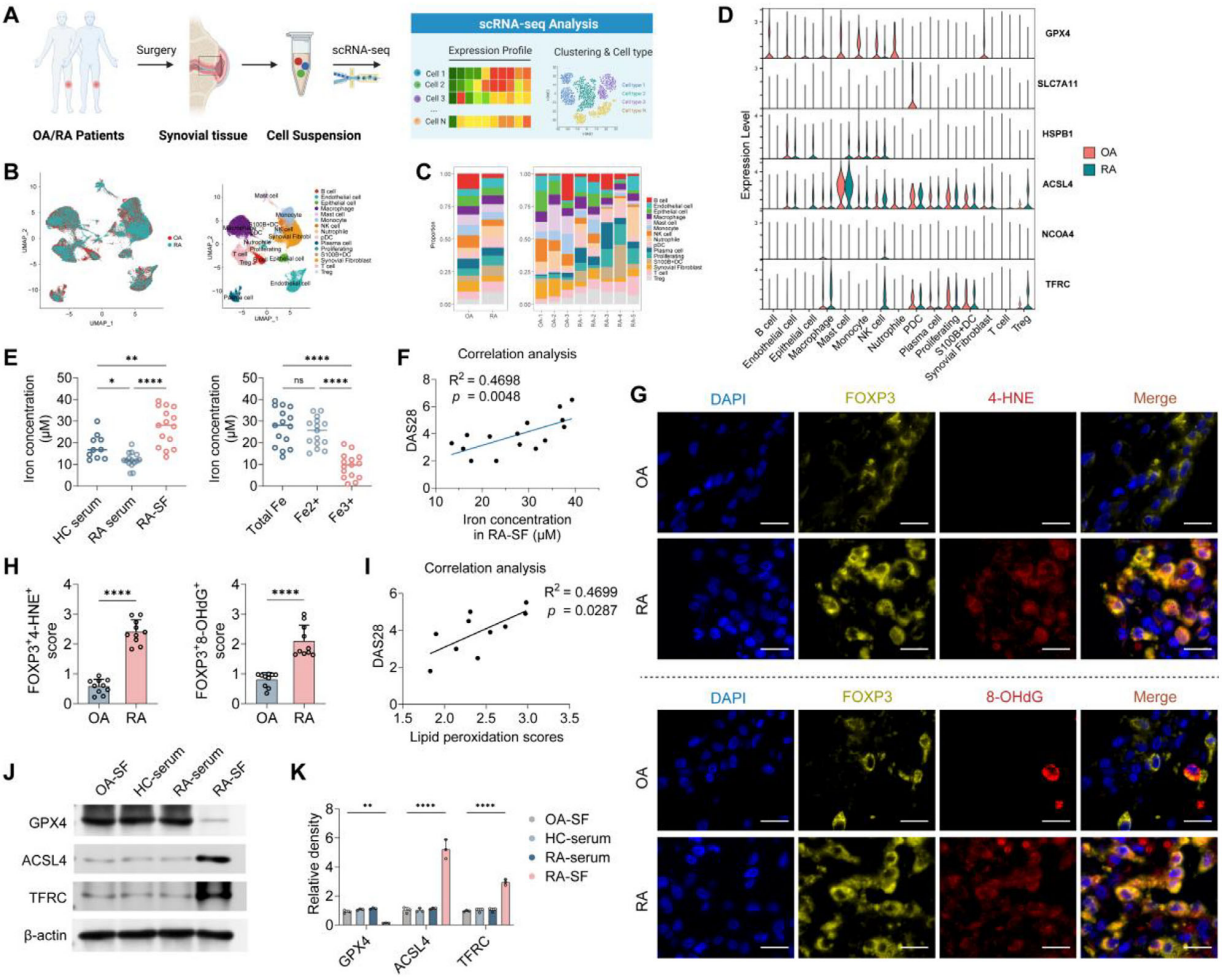
Single‐cell profiling of RA synovial tissue and characterization of ferroptosis in human Tregs. (A) Overview of the study design, comparing RA synovial tissues with high inflammatory infiltration (*n* = 5) to osteoarthritis (OA) tissues with minimal inflammation (*n* = 3) as controls. Matched scRNA‐seq analyses were performed to characterize the cellular landscape of RA synovial samples. (B) Unsupervised clustering analysis identified 14 distinct cell populations, including both immune and stromal cell types, within the synovial tissue. (C) Quantification of the relative distribution of each cell subtype. (D) Gene expression profiles related to ferroptosis across various cell subtypes. (E) Colorimetric assays were conducted to assess free ferrous iron concentrations in RA synovial fluid (*n* = 15), RA serum (*n* = 15), and HC serum (*n* = 10). (F) Statistical analysis investigating the relationship between ferrous iron levels in synovial fluid and the DAS28 in RA patients (*n* = 15). (G,H) Representative images from immunofluorescence staining of 4‐HNE and 8‐OHdG in synovial tissues. Scale bars, 500 µm. (I) Analysis of the correlation between lipid peroxidation levels in Tregs within synovial tissues and the DAS28 score in RA patients. (J,K) Tregs isolated from healthy donor PBMCs were cultured *ex vivo* in the presence of 10% (v/v) OA‐SF, HC‐serum, RA‐serum, or RA‐SF for 48 h, the protein levels of GPX4, ACSL4, and TFRC were detected by Western blot. Data are presented as mean ± SD. *, *p* < 0.05; **, *p* < 0.01; ***, *p *< 0.001; ****, *p* < 0.0001.

RA and osteoarthritis (OA) exhibit distinct phenotypic and pathological features. Histological analysis of RA synovial tissues revealed a heterogeneous composition, characterized by varying degrees of lymphocyte and monocyte infiltration [15]. To characterize the synovial microenvironment in RA, we performed scRNA‐seq on synovial tissues from 5 RA patients with high inflammatory infiltration and 3 OA controls with minimal inflammation (Figure 1A; Figure S1). Unsupervised clustering identified 14 major cell populations, including synovial fibroblasts, endothelial cells, macrophages, monocytes, T cells, and Tregs (Figure 1B). Furthermore, the proportion of Tregs was significantly elevated in RA synovium compared to OA controls (Figure 1C), consistent with prior reports of Treg accumulation in inflamed joints.

Given the established dysregulation of iron metabolism in RA synovium, we assessed cell‐type‐specific vulnerability to ferroptosis. Tregs displayed significantly lower expression of key ferroptosis suppressors compared to other synovial subsets: glutathione peroxidase 4 (GPX4) and heat shock protein family B (small) member 1 (HSPB1) expression was reduced versus mast cell, monocyte, NK cell, and fibroblast (Figure 1D). While ferroptosis markers (acyl‐CoA synthetase long chain family member 4 (ACSL4) and transferrin receptor (TFRC)) were more pronounced in RA‐Tregs than in OA‐Tregs (Figure 1D), suggesting heightened ferroptosis susceptibility within RA synovial Tregs.

Supporting the relevance of iron dysregulation, synovial fluid (SF) from RA patients (RA‐SF) contained significantly higher levels of total iron compared to both RA serum and healthy control serum (HC‐serum), and of which, ferrous iron (Fe^2^^+^) accounted for the majority (Figure 1E). Importantly, ferrous iron levels in RA‐SF positively correlated with clinical disease activity (Disease Activity Score 28, DAS28; *R* ^2^ = 0.6658, *p* < 0.001) (Figure 1F), linking synovial iron overload to RA severity. We then investigated markers of oxidative stress and lipid peroxidation within synovial Tregs. Immunofluorescence revealed significantly elevated levels of lipid peroxidation product 4‐hydroxynonenal (4‐HNE) and the oxidative product 8‐hydroxy‐2′‐deoxyguanosine (8‐OHdG) in RA synovial Tregs compared to OA synovial Tregs (Figure 1G,H). Furthermore, lipid peroxidation levels in RA synovial Tregs progressively increased with higher DAS28 scores (Figure 1I), providing direct evidence that Tregs within the RA synovium experience escalating ferroptosis susceptibility as disease activity worsens.

To further validate the pathogenic impact of the RA microenvironment on Treg ferroptosis susceptibility, we performed an *ex vivo* validation assay. Tregs isolated from healthy PBMCs were cultured with RA‐SF or different controls. Consistent with our observations in patient tissues, exposure to RA‐SF, but not to SF from OA patients (OA‐SF) or HC‐serum or RA‐serum, significantly downregulated the ferroptosis suppressor glutathione peroxidase 4 (GPX4) while upregulating the pro‐ferroptotic proteins acyl‐CoA synthetase long chain family member 4 (ACSL4) and transferrin receptor (TFRC) (Figure 1J,K). In addition to the canonical TFRC/SLC7A11/GPX4 pathway, other regulatory pathways contribute to ferroptosis susceptibility. Re‐analysis of our scRNA‐seq data revealed that synovial Tregs, compared to other synovial cell types such as fibroblasts and endothelial cells, exhibited significantly higher expression of arachidonate 15‐lipoxygenase *(ALOX15)*, a key enzyme in the enzymatic lipid peroxidation pathway. Furthermore, *ALOX15* expression was more pronounced in RA synovial Tregs than in their OA counterparts (Figure S2A–C). This suggests that the ALOX15 pathway may also contribute to the heightened ferroptotic vulnerability of Tregs within the iron‐overloaded RA synovial microenvironment. These findings demonstrate that the synovial‐specific impairment is underpinned by a heightened susceptibility to ferroptosis. The establishment of this ferroptosis‐permissive niche is directly driven by iron overload within the synovial fluid, the level of which correlates with disease severity.

### 2.2 Synovial Fluid From a Collagen‐Induced Arthritis Model (CIA‐SF) Induces Ferroptosis in Murine Tregs

**FIGURE 2:**
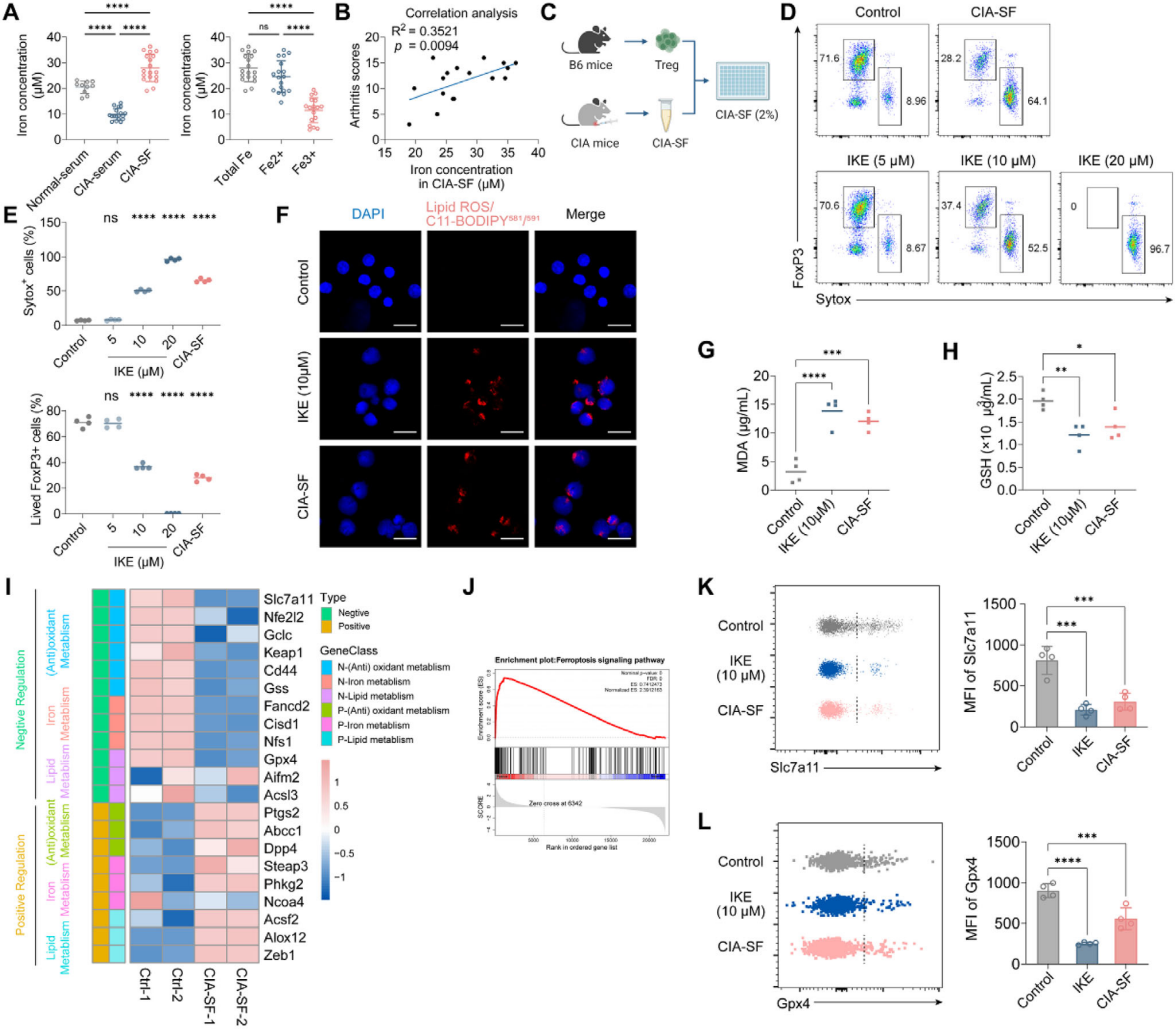
CIA‐SF induces ferroptosis in murine Tregs. (A) Colorimetric assays were conducted to assess total iron and ferrous iron concentrations in CIA‐SF (*n* = 18), CIA‐serum (*n* = 18), and normal serum (*n *= 10). (B) Statistical analysis investigating the relationship between ferrous iron levels in CIA‐SF and the arthritis scores in CIA mice (*n* = 18). (C) Schematic of the experimental design. Tregs isolated from C57BL/6J mice were cultured ex vivo in the presence or absence of 2% (v/v) CIA‐SF or the ferroptosis inducer IKE (positive control) for 24 h. (D,E) Quantification of Treg cell death assessed by SYTOX Green staining. (F) Measurement of intracellular lipid reactive oxygen species (lipid ROS) production using the C11‐BODIPY581/591 probe, analyzed by confocal microscopy. Scale bars, 500 µm. (G,H) Assessment of lipid peroxidation and antioxidant defense: (G) Malondialdehyde (MDA) levels, a marker of lipid peroxidation; (H) Glutathione (GSH) levels, a key antioxidant. (I) Transcriptomic analysis reveals activation of ferroptosis pathways. Tregs were incubated with or without CIA‐SF (2% v/v) for 48 h, followed by RNA sequencing. Heatmap showing differential expression of ferroptosis‐related genes (iron metabolism, lipid metabolism, oxidant metabolism). (J) Gene Set Enrichment Analysis (GSEA) demonstrating significant enrichment of the ferroptosis signaling pathway in CIA‐SF‐treated Tregs. (K,L) CIA‐SF downregulates key ferroptosis defense proteins. Flow cytometric analysis of the mean fluorescence intensity MFI of Slc7a11 and Gpx4 in Tregs. Data are presented as mean ± SD. *, *p* < 0.05; **, *p* < 0.01; ***, *p *< 0.001; ****, *p* < 0.0001.

To further validate the ferroptotic vulnerability of synovial Tregs, we confirmed elevated lipid peroxidation markers (Lipid ROS, 4‐HNE, and malondialdehyde (MDA)) and reduced glutathione (GSH) in Tregs from CIA mice compared to normal mice (Figure S3A–D). We next characterized the synovial fluid from the CIA model. Consistent with the iron overload observed in RA patients, CIA‐SF contained significantly higher levels of total iron and Fe^2^^+^ compared to serum from either CIA mice or normal controls (Figure 2A). Importantly, the concentration of Fe^2^^+^ in CIA‐SF showed a strong positive correlation with the arthritis severity scores in mice (Figure 2B), mirroring our clinical findings and establishing CIA‐SF as a relevant model of the pathologic synovial microenvironment.

Similar to the RA‐SF‐treated human Treg culture system, we established an *ex vivo* model to investigate the effects of CIA‐SF. Purified mouse Tregs were treated with CIA‐SF (2% v/v, concentration optimized by dose‐response) or the ferroptosis inducer imidazole ketone erastin (IKE) for 24 h (Figure 2C). We found CIA‐SF triggered profound ferroptosis, characterized by significantly reduced Treg viability (Figure S3E,F), increased cell death (SYTOX^+^ cells, Figure 2D,E), accumulation of lipid peroxides (C11‐BODIPY^5^ ^8^ ^1^/^5^ ^9^ ^1^ fluorescence, Figure 2F), elevated MDA (Figure 2G), and depletion of the key antioxidant GSH (Figure 2H). Transcriptomic profiling revealed specific activation of ferroptosis pathways in CIA‐SF–treated Tregs. This enrichment was based on observed variations in ferroptosis‐related genes involved in iron metabolism, lipid metabolism, and oxidant metabolism (Figure 2I). Gene Set Enrichment Analysis (GSEA) further showed significant upregulation of ferroptosis signaling pathways in CIA‐SF‐treated Tregs (Figure 2J). CIA‐SF also downregulated core ferroptosis defense genes, evidenced by reduced protein levels of the cystine/glutamate antiporter solute carrier family 7 member 11 (Slc7a11) and the glutathione peroxidase Gpx4 (Figure 2K,L). Collectively, these findings demonstrate that the CIA microenvironment directly induces ferroptosis in Tregs, driven by lipid peroxide accumulation and impaired antioxidant defense mechanisms.​

To confirm the specific role of iron in CIA‐SF‐induced ferroptosis, we used the iron chelator deferoxamine (DFO). Co‐treatment with DFO significantly rescued Tregs from CIA‐SF‐induced cell death (Figure S4A). Furthermore, DFO attenuated the accumulation of lipid ROS and MDA (Figure S4B,C) and restored CIA‐SF‐depleted levels of the key antioxidant GSH (Figure S4D). These data unequivocally demonstrate that iron is a critical driver of ferroptosis in Tregs within the arthritic microenvironment.

### 2.3 A Low Dose of IKE Induces the Phenotypic Changes and Dysfunction in Murine Tregs

**FIGURE 3:**
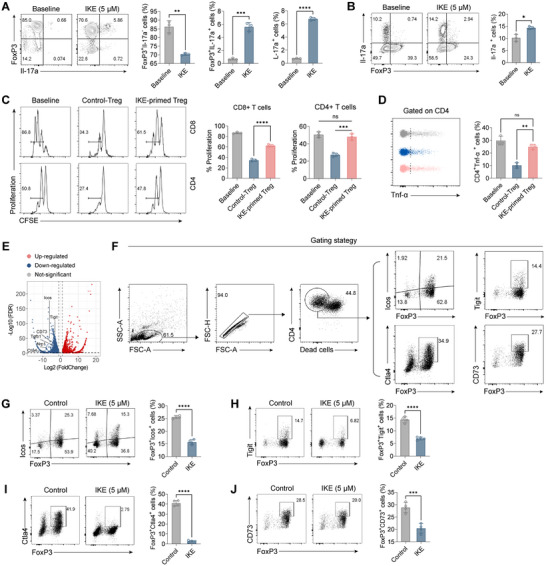
Exposure to a low dose of IKE induces Treg instability and functional impairment. (A,B) A low dose of IKE (5 µm) promotes Treg instability and acquisition of a Th17‐like phenotype. (A) Tregs isolated from C57BL/6J mice were incubated with or without a low dose of IKE for 5 days under Treg culture conditions (100 U/mL Il‐2, and 1:5 mouse T‐Activator CD3/CD28 Dynabeads). Flow cytometry analysis shows the percentage of FoxP3+ Tregs co‐expressing Il‐17a. (B) Under Th17‐polarizing conditions (Il‐6, Tgf‐β, anti‐Ifnγ, anti‐Il‐4), a low dose of IKE exposure significantly enhanced the conversion of Foxp3+ Tregs into Il‐17a+ cells. Representative flow cytometry plots and summarized data are shown. (C,D) A low dose of IKE (5 µm) compromises Treg suppressive function. (C) The capacity of a low dose of IKE‐pretreated Tregs (IKE pretreatment for 24 h) to suppress the proliferation of co‐cultured effector T cells (Teffs, labeled with CFSE and stimulated with anti‐CD3/APCs) was assessed by CFSE dilution analysis. Representative flow cytometry histograms and summarized suppression of proliferation are shown. (D) A low dose of IKE‐pretreated Tregs (IKE pretreatment for 24 h) exhibited diminished capacity to suppress Tnf‐α production by Teffs, analyzed by intracellular cytokine staining and flow cytometry. (E) Transcriptomic analysis (RNA‐seq) of Tregs exposed ex vivo to a low dose of IKE (5 µm, 48 h) showed significant downregulation of genes critical for Treg function and stability (Icos, Tigit, Ctla4, CD73/Eno1, Nrp1, and Il10) compared to untreated controls. (F–J) Flow cytometric validation at the protein level confirmed significant downregulation of (G) Icos, (H) Tigit, (I) Ctla4, and (J) CD73 in Tregs exposed to a low dose of IKE (5 µm, 48 h). (See Figure 4SA, B for Nrp1 and Il‐10). Data are presented as mean ± SD. *, *p* < 0.05; **, *p* < 0.01; ***, *p *< 0.001; ****, *p* < 0.0001.

Having established that CIA‐SF induces ferroptosis in Tregs, we next investigated whether this ferroptotic stress compromises the phenotypic stability and suppressive function of the surviving Tregs. When purified mouse Tregs were exposed to a low dose of IKE (5 µm) for 5 days, they underwent a profound shift toward a Th17‐like phenotype. Specifically, this low‐dose IKE treatment caused a dramatic increase in the frequency of Il‐17a^+^ Tregs under standard culture conditions (from 0.74% to 6.5%, *p* < 0.0001; Figure 3A). This proinflammatory skewing was markedly amplified under Th17‐polarizing conditions, where this low dose of IKE significantly enhanced the conversion of FoxP3^+^ Tregs into pathogenic Il‐17a^+^ cells (Figure 3B). Concomitant with this phenotypic instability, the low dose of IKE severely compromised the core suppressive function of Tregs. Pretreatment with the low dose of IKE significantly impaired the ability of Tregs to inhibit the proliferation of co‐cultured effector T cells (Teffs), as evidenced by reduced CFSE dilution (Figure 3C). Furthermore, the low dose of IKE‐pretreated Tregs exhibited diminished capacity to suppress Tnf‐α cytokine production by Teffs (Figure 3D), underscoring a broad functional deficit.

Transcriptomic profiling also revealed the molecular underpinnings of this dysfunction. The low dose of IKE exposure induced a global downregulation of genes essential for Treg identity and function, including co‐stimulatory molecules (Icos, Tigit), key suppressive mediators (Tgfb1), and surface receptors critical for stability and suppressive capacity (Ctla4, Nrp1, CD73; Figure 3E). Flow cytometric analysis confirmed this downregulation at the protein level, demonstrating significantly reduced expression of Icos (*p* < 0.0001), Tigit (*p* < 0.0001), Ctla‐4 (*p* < 0.001), CD73 (*p* < 0.001), Neuropilin‐1 (Nrp1, *p* < 0.001) and Il‐10 (*p* < 0.001) in this low dose of IKE‐exposed Tregs (Figure 3F–J; Figure S5A,B). These results establish that ferroptosis stress actively destabilizes Tregs, crippling their immunosuppressive machinery through the downregulation of critical functional molecules.​

### 2.4 Ferroptosis‐Sensitive Tregs are Associated With Severe Changes in OXPHOS

**FIGURE 4:**
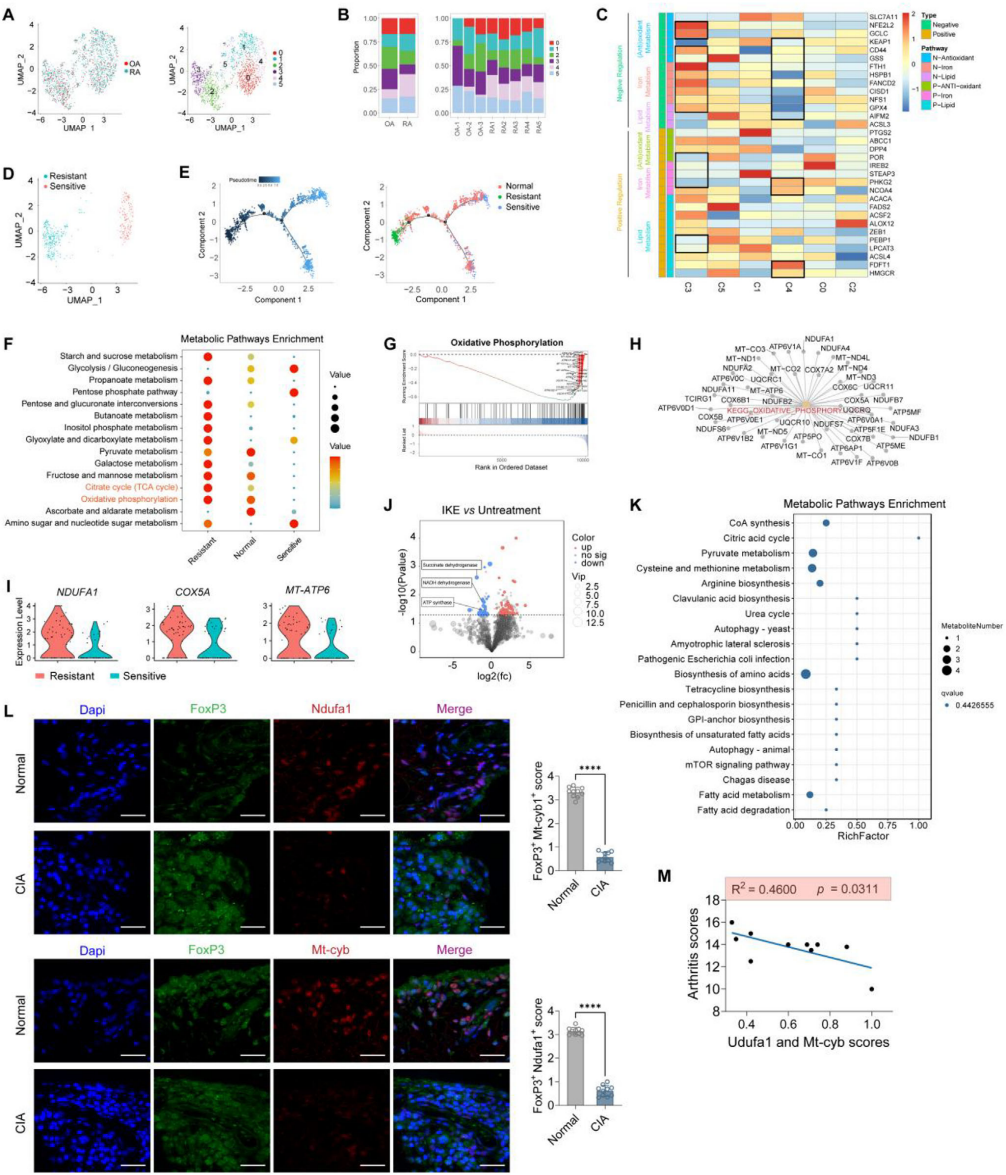
Ferroptosis‐sensitive Tregs exhibit severe alterations in oxidative phosphorylation in the RA microenvironment. (A) Uniform Manifold Approximation and Projection (UMAP) was applied to visualize Treg populations, identifying six distinct clusters based on scRNA‐seq data. (B) The relative proportions of each Treg cluster across different samples were determined from the scRNA‐seq dataset. (C,D) Treg clusters were classified into ferroptosis‐resistant and ferroptosis‐sensitive subsets according to the expression of high‐risk ferroptosis genes in the scRNA‐seq analysis. (E) Pseudotime analysis of ferroptosis‐resistant and ferroptosis‐sensitive Treg subsets was performed to track their developmental trajectory within the scRNA‐seq data. (F) Enrichment plots from KEGG pathway analysis were used to compare the pathways enriched in ferroptosis‐resistant versus ferroptosis‐sensitive Treg subsets in scRNA‐seq. (G) GSEA was conducted on the ferroptosis‐sensitive Treg subset, providing insights into enriched pathways within this population. (H) Network analysis of gene expression revealed that OXPHOS signaling is a major functional category, showing reduced gene expression in ferroptosis‐sensitive Tregs from the scRNA‐seq dataset. (I) Expression levels of key ETC‐associated genes (NDUFA1, COX5A, and MT‐ATP6) were compared between ferroptosis‐resistant and ferroptosis‐sensitive Tregs using scRNA‐seq data. (J) LC‐MS was employed to analyze metabolite alterations in Tregs cultured with or without 10 µm IKE for 48 h. (K) Functional enrichment analysis of Tregs cultured with or without 10 µm IKE for 48 h using LC‐MS. (L) Representative immunofluorescence images of mitochondrial respiratory chain components, NDUFA1 and MT‐CYB, in Tregs from CIA mice. Scale bars, 200 µm. (M) Correlation analysis between the expression levels of NDUFA1 and MT‐CYB and the severity of arthritis as assessed by activity scores. In M, data are presented as mean ± SEM, *n* = 5–8 mice. ****, *p *< 0.0001.

We sought to dissect the metabolic basis underlying the heightened ferroptotic susceptibility of synovial Tregs by re‐clustering Tregs from the RA synovium in our scRNA‐seq dataset. The UMAP visualization revealed six distinct Treg clusters (Figure 4A,B). These clusters were stratified into ferroptosis‐resistant and sensitive subsets by pathway activity analysis, which hinged on the differential expression of genes governing iron handling, lipid metabolism, and redox balance (Figure 4C,D). The pseudotemporal trajectory analysis revealed that the resistant Treg state and the sensitive state showed continuity at the transcriptome level, suggesting that in the inflammatory microenvironment, Tregs may undergo a functional state transition from resistance to sensitivity. However, this requires future lineage tracing studies to be directly verified (Figure 4E). Furthermore, ferroptosis‐sensitive Tregs exhibited profound metabolic alterations. scRNA‐seq data revealed significant downregulation of OXPHOS and tricarboxylic acid (TCA) cycle pathways within this subset (Figure 4F). Consistent with these findings, GSEA indicated that OXPHOS was downregulated in ferroptosis‐sensitive Tregs (Figure 4G). Gene co‐expression network analysis further identified OXPHOS signaling as the primary downregulated functional module (Figure 4H). Consistently, the key mitochondrial respiratory chain components, including NADH: ubiquinone oxidoreductase subunit A1 (NDUFA1, Complex I), cytochrome c oxidase subunit 5A (COX5A, Complex IV), and mitochondrially encoded ATP synthase 6 (MT‐ATP6, Complex V) were significantly reduced expression in ferroptosis‐sensitive Tregs (Figure 4I).

Functional metabolic profiling corroborated these transcriptional signatures. Untargeted metabolomics of Tregs exposed *ex vivo* to IKE demonstrated significant perturbations in mitochondrial metabolism. Enzymes critical for OXPHOS flux were dysregulated, including succinate dehydrogenase (SDH; Complex II), NADH dehydrogenase (Complex I), and ATP synthase (Complex V) (Figure 4J). Pathway analysis confirmed severe disruption of core energy pathways, notably the TCA cycle and CoA synthesis (Figure 4K). These metabolic defects in Tregs were further validated using an established experimental arthritis model. Immunofluorescence analysis of Tregs within joints from CIA mice showed significantly reduced protein expression of Ndufa1 and Mt‐cyb compared to normal mice (Figure 4L). Furthermore, the expression scores of Ndufa1 and Mt‐cyb inversely correlated with arthritis severity scores, establishing a direct link between Treg mitochondrial dysfunction and arthritis disease progression (Figure 4M). Thus, synovial Tregs display functional heterogeneity in their metabolic adaptation to the arthritic joint, with ferroptosis‐sensitive subsets undergoing specific reprogramming characterized by collapse of mitochondrial OXPHOS and TCA cycle activity—a vulnerability intrinsically linked to their susceptibility to ferroptosis.​

### 2.5 CIA‐SF‐Induced Ferroptosis Drives Mitochondrial ETC Damage and Metabolic Crisis in Murine Tregs

**FIGURE 5:**
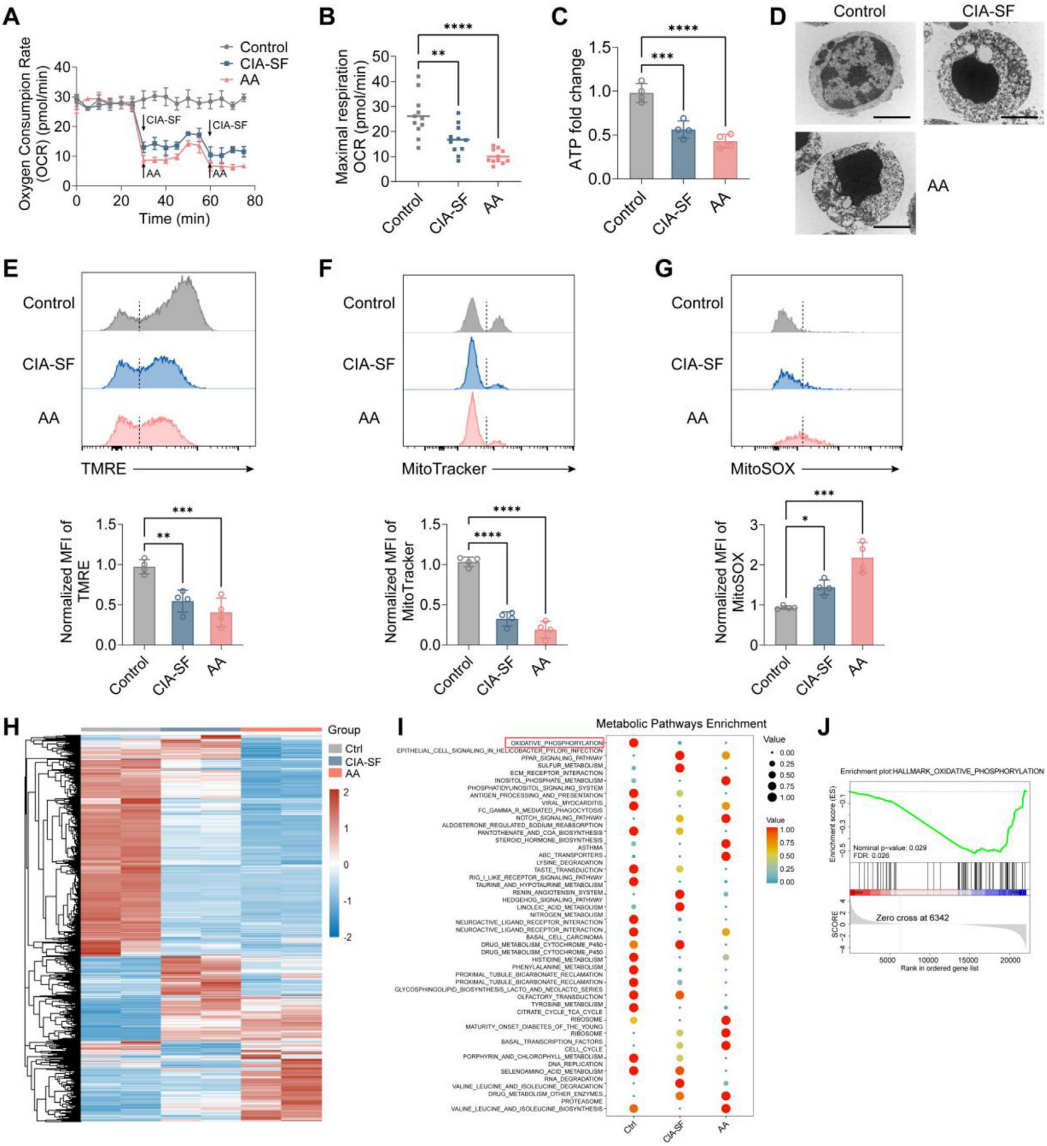
Ferroptotic stress or direct ETC inhibition induces mitochondrial dysfunction in Tregs. (A,B) Tregs were treated with CIA‐SF (2% v/v) or the mitochondrial complex III inhibitor antimycin A (AA, 10 µm, positive control). Mitochondrial respiration was assessed using the Seahorse XF Analyzer. (A) Overlay of real‐time oxygen consumption rate (OCR) traces. Arrows indicate sequential treatment of CIA‐SF or AA during the mitochondrial stress test. (B) Quantification of maximum respiratory capacity. (C) Tregs were exposed with CIA‐SF or AA for 12 h, ATP content was relatively quantified. (D) Ultrastructural evidence of mitochondrial damage. Representative transmission electron microscopy (TEM) images of Tregs treated with CIA‐SF or AA (10 µm, 12 h). Scale bars, 2 µm. (E–G) Functional impairments in mitochondrial integrity. (E) Significant decrease in mitochondrial membrane potential assessed by TMRE fluorescence (flow cytometry) after 12 h exposure to CIA‐SF or AA (10 µm). (F) Reduction in mitochondrial mass measured by MitoTracker Red fluorescence (flow cytometry) in Tregs exposed to CIA‐SF or AA (10 µm, 12 h). (G) Increase in mitochondrial ROS production detected by MitoSOX Red fluorescence (flow cytometry) in Tregs exposed to CIA‐SF or AA (10 µm, 12 h). (H–J) Tregs were exposed to CIA‐SF (2% v/v) or AA (10 µm) for 24 h. RNA‐seq and GSEA identified the OXPHOS pathway as significantly downregulated in Tregs in the presence of CIA‐SF. In (A–G), data are shown as mean ± SD. *, *p* < 0.05; **, *p* < 0.01, ***, *p *< 0.001; ****, *p *< 0.0001.

Lipid peroxidation drives ferroptosis and disrupts mitochondrial integrity [16, 17]. To test whether CIA‐SF‐induced ferroptosis impairs the mitochondrial ETC in Tregs, we assessed their respiratory function. CIA‐SF treatment significantly reduced both the oxygen consumption rate (OCR) and the maximal respiratory capacity of Tregs (Figure 5A,B). Consequently, intracellular ATP levels were substantially depleted (Figure 5C). This bioenergetic deficit, comparable to the effect of the complex III inhibitor Antimycin A (AA), establishes a direct link between CIA‐SF‐induced ferroptosis and respiratory failure. Electron microscopy further confirmed severe ultrastructural damage in CIA‐SF‐exposed Tregs, characterized by cytoplasmic condensation, disorganized and reduced mitochondrial cristae, and outer mitochondrial membrane disruption (Figure 5D). These morphological alterations were corroborated by functional deficits. Specifically, tetramethylrhodamine ethyl ester (TMRE) staining showed a marked loss of mitochondrial membrane potential (Figure 5E). Moreover, a decrease in mitochondrial mass (MitoTracker) and a concomitant increase in mitochondrial ROS (MitoSOX) were observed (Figure 5F,G), confirming profound mitochondrial dysfunction.

Transcriptomic analysis provided molecular evidence for the observed mitochondrial dysfunction. RNA‐seq revealed significant downregulation of the OXPHOS pathway in CIA‐SF‐treated Tregs (Figure 5H,I). GSEA further confirmed diminished enrichment of OXPHOS‐related genes (Figure 5J), aligning perfectly with the metabolic signature of ferroptosis‐sensitive synovial Treg subsets identified earlier (Figure 4F–I). These results suggest that CIA‐SF‐induced lipid peroxidation causes direct structural and functional damage to the mitochondrial ETC. This injury manifests as impaired respiration, collapsed membrane potential, ATP depletion, and ultrastructural degeneration, culminating in a profound metabolic crisis that drives Treg dysfunction.

To define the specific metabolic lesions underlying the observed OXPHOS collapse, we performed isotope‐tracing and substrate‐dependency assays. ^13^C‐palmitate​ tracing revealed a severe and specific impairment in fatty acid oxidation (FAO)​ in CIA‐SF/IKE‐treated Tregs. The incorporation of palmitate‐derived 13C into key TCA cycle intermediates (citrate, α‐ketoglutarate, succinate, malate) revealed markedly lower ^13^C enrichment in CIA‐SF/IKE‐treated Tregs compared to control Tregs (Figure S6A,B), indicating a near‐complete loss of oxidative capacity for fatty acids. Intriguingly, parallel tracing with U‐^13^C‐glucose​ showed a different picture. Glucose oxidation flux, measured by ^13^C enrichment in TCA intermediates, was modestly elevated​ in CIA‐SF/IKE‐treated Tregs (Figure S6C,D). This suggests a compensatory, yet insufficient, shift toward glucose utilization in the face of FAO failure. This metabolic rewiring—catastrophic loss of FAO coupled with inefficient glucose oxidation—was confirmed in real‐time by a Seahorse XF Substrate Dependency Test. Sequential pharmacological inhibition of glucose oxidation (using 2‐deoxyglucose, 2‐DG) and FAO (using etomoxir) pinpointed the relative contribution of each pathway. The basal OCR of CIA‐SF/IKE‐treated Tregs was exquisitely sensitive to etomoxir​ but only minimally affected by 2‐DG (Figure S6E,F), demonstrating their profound reliance on, and vulnerability due to, the failing FAO pathway. The residual, 2‐DG‐insensitive respiration likely represents an insufficient basal activity or alternative fuel use.

Collectively, these data establish that ferroptosis susceptibility in synovial Tregs is not due to a general metabolic quiescence but is specifically linked to a critical breakdown in FAO. This defect creates an irreparable bioenergetic deficit by depriving the ETC of a major reducing equivalent source, when FAO is inhibited, Tregs revert to glucose dependence, often losing function.

### 2.6 Inflammatory Reprogramming in Ferroptotic Tregs Is Driven by TXK via STAT3 and PLCγ1 Phosphorylation

**FIGURE 6:**
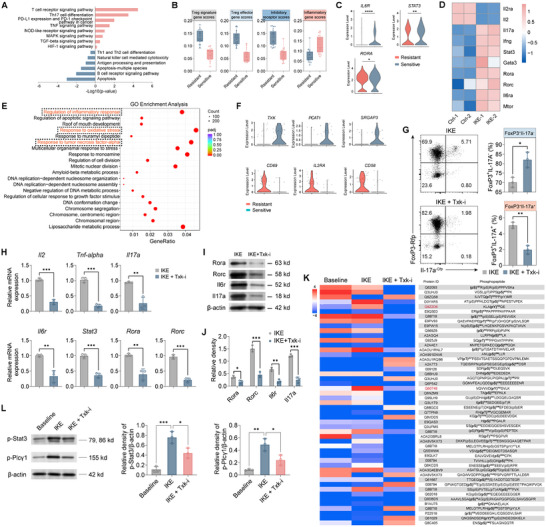
TXK activates STAT3/PLCγ1 to drive inflammation‐prone Treg phenotype. (A) Enrichment histogram from KEGG pathway analysis among ferroptosis‐resistant and ferroptosis‐sensitive Treg subsets by scRNA‐seq. The red bars represent terms highly enriched in the ferroptosis‐sensitive Treg subset, while blue bars represent terms enriched in the ferroptosis‐resistant subset. (B) Gene set scores for Treg signature genes, effector molecules, inhibitory receptors, and inflammatory markers across ferroptosis‐resistant and ferroptosis‐sensitive Treg subsets, as analyzed by scRNA‐seq. (C) Expression levels of Treg signature genes or inflammatory markers (*IL6R*, *STAT3*, and *RORA*) in ferroptosis‐resistant and ferroptosis‐sensitive Tregs, as measured by scRNA‐seq. (D) Expression of inflammation‐associated genes in Tregs exposed with or without IKE (5 µm) for 48 h, analyzed by RNA‐seq. (E) Functional enrichment analysis of IKE‐treated Tregs, based on RNA‐seq data of D. (F) Expression levels of genes identified in ferroptosis‐resistant and ferroptosis‐sensitive Treg subsets as determined by scRNA‐seq. (G–L) Murine Tregs were treated with IKE (5 µm) in the presence or absence of a Txk inhibitor (Txk‐i, 10 nm, HY‐10644, MCE) for 48 h. (G) Phenotypic changes of Tregs into IL‐17a+ Th17‐like cells were assessed by flow cytometry. (H) The relative mRNA expression levels of inflammatory hallmarks (*Tnf‐α*, *Ifng*, *Il17a*, *Il6r*, *Stat3*, *Rora*, and *Rorc*) were measured by qPCR. (I,J) The relative protein levels of Rora, Rorc, Il6r, and Il‐17a were measured by Western blot. (K) Phosphoproteomic analysis via LC‐MS.​ (L) The relative protein levels of p‐Stat3 and p‐Plcγ1 were measured by Western blot. Data are shown as mean ± SD. *, *p* < 0.05; **, *p* < 0.01, ***, *p *< 0.001; ****, *p *< 0.0001.

We began by characterizing the ferroptosis‐sensitive Treg subset (initially defined in Figure 4D). KEGG enrichment analysis indicated a significant association of this subset with pathways involved in Th17 cell differentiation and TNF signaling (Figure 6A), suggesting that an activation of inflammatory signaling may represent a crucial event initiated by ferroptosis in Tregs. Consistent with this functional prediction, ferroptosis‐sensitive Tregs displayed a pronounced shift toward an inflammatory phenotype. Gene set scoring demonstrated a significant reduction in core Treg signature genes (*CD25*, *FOXP3*, *ICOS*, *HELIOS*), suppressive effector molecules (*IL‐10*, *TGF‐β*), and inhibitory receptors (*CTLA4*, *TIM3*, *LAG3*, *NRP1*, *GALECTIN‐1*, *CD39*, *CD73*). Conversely, genes associated with inflammation (*IFN‐γ*, *TNF‐α*, *IL‐6R*, *STAT3*, *RORA*, *RORC*, *IL‐17A*, *MTOR*, *IRF4*, *GATA3*) were significantly upregulated (Figure 6B). Key examples of this dysregulation, including elevated *IL6R*, *STAT3*, *RORA*, are visualized in the violin plot (Figure 6C). We next asked if ferroptotic stress alone could drive this inflammatory reprogramming. Treating Tregs with a low dose of IKE recapitulated the signature: mRNA levels of *Il17a*, *Ifng*, *Stat3*, and other inflammatory genes were increased, while *Il2ra (CD25)* and *Il2* were decreased (Figure 6D). Functional enrichment analysis confirmed the upregulation of inflammatory responses, TNF‐α signaling, and oxidative stress responses (Figure 6E).

Differential expression analysis displayed a distinct marker profile in ferroptosis‐sensitive Treg compared to their resistant counterparts, with elevated transcripts for *TXK*, *PCAT1*, and *SRGAP3*, but reduced expression of *CD69*, *IL2RA* (CD25), and *CD58* (Figure 6F). Given the prominent upregulation of the tyrosine kinase TXK in the sensitive subset, and its known role in Th1 responses and previously associated with RA [18, 19], we found that both IKE and the mitochondrial inhibitor AA significantly induced TXK protein expression (Figure S7A,B). We next sought to determine the upstream signal connecting ETC damage to TXK upregulation. As both IKE and AA treatment robustly increased mitochondrial ROS (mtROS, Figure 5G), we hypothesized that mtROS acts as a critical second messenger in this pathway. To test this, we co‐treated Tregs with IKE and the mitochondrial‐targeted antioxidant Mito‐TEMPO. Scavenging mtROS effectively blocked IKE‐induced TXK upregulation (Figure S7C,D), establishing mtROS as a necessary signal linking ETC dysfunction to TXK induction. This positions TXK as a metabolic stress sensor activated by ferroptosis‐associated mitochondrial damage.

To define TXK's functional role, we used a pharmacological inhibitor (Txk‐i). Txk‐i significantly attenuated the IKE‐induced conversion of Foxp3+ Tregs into pathogenic Il‐17a+ cells (Figure 6G) and suppressed the upregulation of key inflammatory markers (*Tnf‐α*, *Ifng*, *Il17a*, *Il6r*, *Stat3*, *Rora*, *Rorc*) at the mRNA (Figure 6H) and protein levels of Rroa, Rorc, Il6r, and Il‐17a (Figure 6I,J). Having established the critical role of TXK in driving the inflammatory shift in ferroptotic Tregs, we further investigated the downstream signaling mechanisms. Phosphoproteomic profiling revealed that, the phosphorylation of Stat3 at the critical tyrosine 705 residue (Q9Z2D6, Stat3‐Y705) and phosphorylation of Plcγ1 at the tyrosine 783 residue (Q60748, Plcγ1‐Y783), essential for its transcriptional activity, were prominently modulated by IKE, directly linking ferroptosis activity to the functional activation of inflammatory signaling. While Txk inhibition globally altered the phosphorylation landscape (Figure 6K). Further investigation indicates that Txk‐i partially abrogated IKE‐induced phosphorylation of STAT3 and PLCγ1, providing strong evidence that TXK is necessary for activating the STAT3/PLCγ1 axis under ferroptotic stress conditions (Figure 6L). In summary, ferroptotic stress induces TXK expression, which in turn phosphorylates and activates both STAT3 and PLCγ1, thereby driving the pro‐inflammatory transcriptional reprogramming that underlies Treg dysfunction.

### 2.7 Targeting Ferroptosis Restores Treg Function and Ameliorates Experimental Arthritis

**FIGURE 7:**
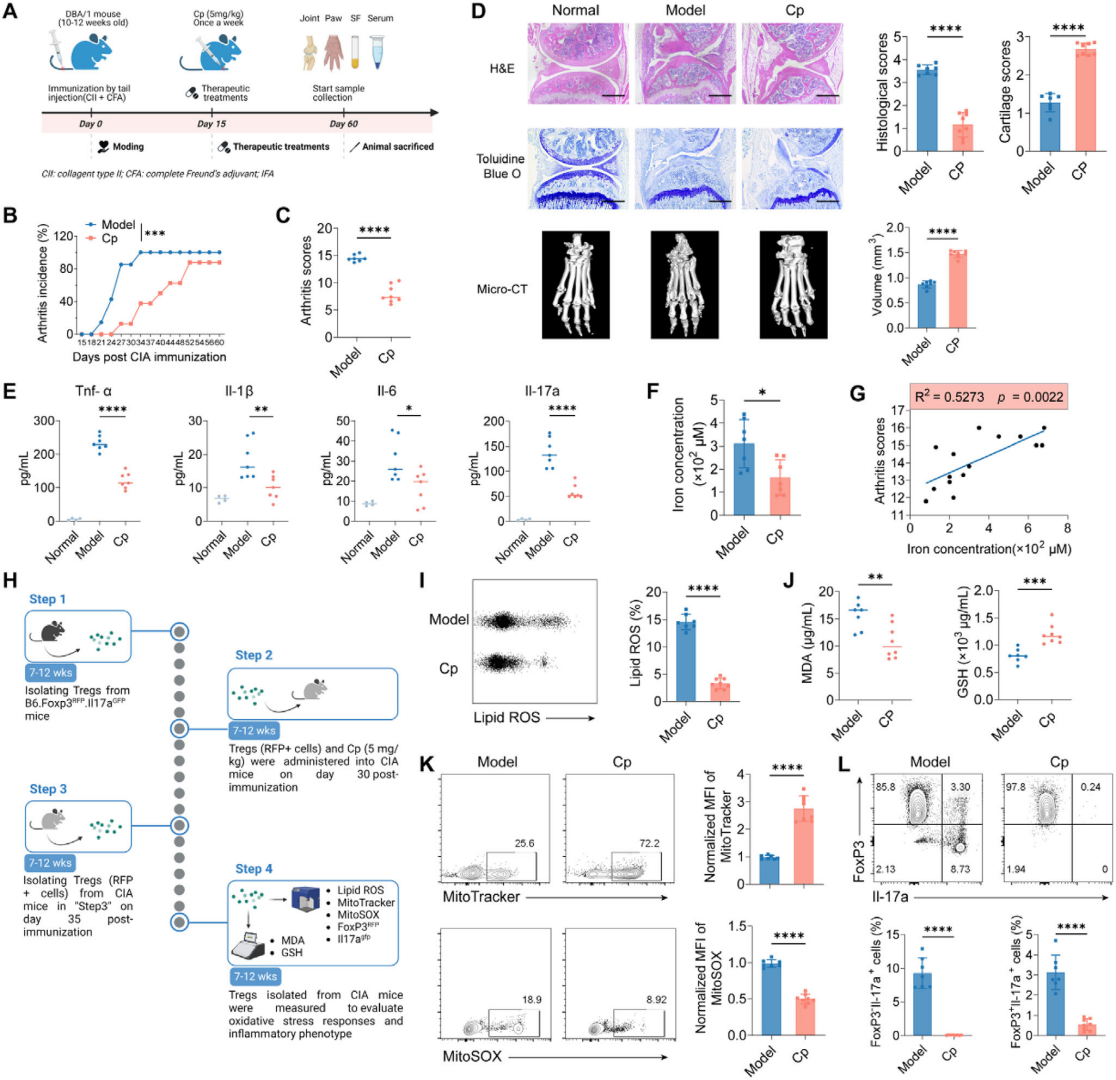
Ceruloplasmin (Cp) mitigates Treg dysfunction and mitigates CIA progression through modulation of ferroptosis. (A) The experimental design outlines the CIA model and the administration of Cp (5 mg/kg via intra‐articular injection). Treatment began on day 15 post‐immunization, with injections repeated weekly until day 50 post‐immunization in CIA mice. (B) The incidence of arthritis in CIA mice was monitored from day 15 to day 60 following immunization. (C) The arthritis severity scores of CIA mice were evaluated on day 60 after immunization. (D) Histological evaluation was performed to assess synovial hyperplasia, inflammation, and cartilage damage in the joints, along with micro‐CT analysis to evaluate bone erosion in the paws. Scale bars, 500 µm. (E) Serum cytokine levels (Tnf‐α, Il1β, Il6, and Il17a). (F) Analysis of ferrous iron levels in synovial fluid. (G) A correlation between synovial iron concentration and clinical arthritis scores was determined using linear regression (*R* ^2^ = 0.5273, *p* < 0.01). (H) Schematic for (H–K): on day 30 post‐immunization in CIA mice, Tregs (isolated from B6.Foxp3^RFP^.Il17a^GFP^ mice, via intravenous injection) and Cp (5 mg/kg via intra‐articular injection) were administered in CIA mice. On day 35 post‐immunization, mice were euthanized, and exogenously injected FoxP3^RFP^+ Tregs were isolated from the spleen tissue of CIA mice for subsequent analysis of (H–K). (I) Assessment of lipid ROS production in Tregs. (J) Measurement of lipid peroxide MDA and antioxidant GSH levels in Tregs. (K) Evaluation of mitochondrial dynamics and ROS production in Tregs, MitoTracker, and MitoSOX MFI values were normalized to model mice. (L) The conversion of Tregs into pathogenic Th17 cells was analyzed through flow cytometry. Data are presented as mean ± SEM, *n* = 5–8 mice. *, *p* < 0.05; **, *p* < 0.01; ***, *p *< 0.001; ****, *p *< 0.0001.

To evaluate the therapeutic potential of mitigating synovial ferroptotic stress, we targeted synovial iron overload using ceruloplasmin (Cp). Cp is an extracellular ferroxidase that catalyzes the oxidation of Fe^2+^ to Fe^3+^, promoting iron efflux and inhibiting ferroptosis [20]. We administered Cp locally via intra‐articular injection (5 mg/kg) to mice with CIA mice, beginning at disease onset (day 15 post‐immunization) and repeating weekly (Figure 7A). Cp treatment significantly ameliorated arthritis severity. It delayed disease onset (Figure 7B), attenuated joint swelling (Figure S8A), and reduced clinical arthritis scores (Figure 7C; Figure S8B). Histopathological analysis confirmed substantial improvements: Cp‐treated mice exhibited markedly reduced synovial hyperplasia, inflammatory cell infiltration, cartilage destruction, and bone erosion compared to vehicle‐treated controls (Figure 7D). Micro‐CT analysis further corroborated reduced bone damage. Concomitantly, serum levels of key pro‐inflammatory cytokines (Tnf‐α, Il‐1β, Il‐6, Il‐17a) were significantly lower in Cp‐treated mice (Figure 7E). Notably, Cp effectively corrected synovial iron dyshomeostasis. As anticipated, intra‐articular Cp significantly reduced Fe^2+^ levels in the SF (Figure 7F). Furthermore, Fe^2+^ concentration in the SF remained strongly correlated with clinical arthritis severity (*R* ^2^ = 0.5273, *p *< 0.01; Figure 7G), reinforcing the link between local iron overload and disease progression.

To directly assess the impact of Cp on synovial Treg functionality in vivo, we employed an adoptive transfer model. On day 30 post‐immunization (moderate immune response), CIA mice received intravenous injection of Tregs isolated from B6.Foxp3^RFP^.Il17a^GFP^ reporter mice, combined with intra‐articular Cp or vehicle (Figure 7H). Five days later (on day 30 post‐immunization), transferred FoxP3^RFP^+ Tregs were isolated from the spleen (reflecting systemic recirculation of synovium‐exposed cells) for analysis. Cp treatment protected Tregs from ferroptosis and restored mitochondrial integrity. Compared to Tregs isolated from vehicle‐treated CIA mice, those from Cp‐treated mice exhibited significantly reduced levels of lipid peroxidation markers (lipid ROS and MDA) and elevated GSH (Figure 7I,J). Furthermore, Cp improved mitochondrial quality, as evidenced by increased mitochondrial mass (MitoTracker) and decreased mitochondrial ROS production (MitoSOX) (Figure 7K). Crucially, mitigating ferroptotic stress via Cp prevented the pathogenic inflammatory reprogramming of Tregs. Flow cytometric analysis revealed a significantly lower frequency of FoxP3^RFP^+ Tregs that had converted to Il‐17a^GFP^+ (Th17‐like) cells in Cp‐treated mice compared to vehicle controls (Figure 7L). These results demonstrate that targeted inhibition of synovial ferroptosis effectively preserves Treg mitochondrial function, prevents their inflammatory transformation, restores Treg‐mediated immune regulation, and ultimately ameliorates experimental arthritis.

## 3 Discussion

​This study delineates a novel and pivotal pathogenic cascade in RA: synovial iron overload establishes a ferroptosis‐permissive microenvironment that specifically targets Tregs, inducing mitochondrial ETC damage. This metabolic crisis, in turn, drives TXK kinase‐dependent activation of STAT3 (Tyr705) and PLCγ1 (Tyr783), culminating in the inflammatory reprogramming of Tregs into dysfunctional Tregs. This mechanism fundamentally resolves the long‐standing paradox of abundant yet functionally inert synovial Tregs in RA and identifies TXK signaling as a master regulator of Treg stability under metabolic stress. Our findings extend beyond the established link between synovial iron/ferroptosis and stromal cell/joint damage [7, 8, 21, 22], by pinpointing Treg dysfunction as a critical immune tolerance checkpoint disrupted by this microenvironmental trigger.​

Ferroptosis is an iron‐dependent cell death driven by the lethal accumulation of lipid peroxides [10, 23]. The key processes underlying ferroptosis involve the depletion of GSH and a reduction in the activity of GPX4, which impairs the detoxification of lipid peroxides through GPX4‐catalyzed reactions. As a result, the oxidation of ferrous ions promotes lipid peroxidation, thereby triggering ferroptosis [9, 23, 24]. Recent studies have highlighted the pivotal role of ferroptosis in autoimmune diseases. This iron‐dependent cell death may exacerbate the inflammatory response and contribute to joint destruction in RA [7, 8, 21, 22]. Ferroptosis is increasingly implicated in RA pathogenesis. Regulators of ferroptosis may hold significant therapeutic potential in both preclinical and clinical settings. While the role of ferroptosis in the pathogenesis of RA is gaining attention, its precise mechanisms remain poorly understood. While previous work linked ferroptosis to synovial fibroblasts [25], macrophages [26], and chondrocytes [22], we demonstrate that synovial Tregs possess intrinsic susceptibility to ferroptosis, evidenced by scRNA‐seq revealing deficient expression of key protective genes (GPX4 and HSPB1). Cross‐species experiments shown that exposure to CIA‐SF, which recapitulates the iron‐overloaded synovial milieu, is sufficient to induce ferroptosis in Tregs. This process is characterized by lethal lipid peroxide accumulation, failure of antioxidant defenses (downregulation of Slc7a11 and Gpx4), and mitochondrial damage. In addition to these findings, we confirmed that CIA‐SF‐induced ferroptosis stress perturbs Tregs’ phenotypic changes and functional molecules expression. This selective sensitivity provides a mechanistic basis for the preferential impairment of Tregs within the inflamed joint, despite their numerical accumulation.​

The profound impact of ferroptosis on Tregs extends beyond cell death to encompass a fate‐altering reprogramming of surviving cells. Our data establish that ferroptosis or lipid peroxide accumulation triggered by synovial fluid inflicts severe damage to mitochondrial ETC components (evidenced by reduced NDUFA1/Complex I, COX5A/Complex IV, MT‐ATP6/Complex V), leading to structural disorganization (cristae loss), and increased mtROS in Tregs. This study identifies mitochondrial ETC dysfunction not merely as a consequence of ferroptosis, but as the essential metabolic crisis trigger for the subsequent pathogenic signaling reprogramming in surviving Tregs. Mounting evidence implicates dysregulation of Treg metabolism can lead to a breakdown in immune tolerance and contribute to the development of autoimmune diseases [27, 28]. Previous studies have shown that, in contrast to effector T cells which primarily rely on glycolysis, the functional integrity of Tregs is particularly dependent on OXPHOS [29, 30, 31]. Consistent with this, our data demonstrate that within the articular synovial microenvironment, the OXPHOS pathway in Tregs is specifically impaired, which is associated with their unique susceptibility to ferroptosis.

Our integrated metabolic profiling, combining isotopic flux analysis, substrate dependency testing, and OCR measurement, reveals that CIA‐SF/IKE‐treated Tregs suffer from a specific and profound lesion in FAO utilization. These cells cannot efficiently channel carbon from fatty acids through the TCA cycle, leading to a substrate‐level starvation of the ETC. This specific metabolic lesion—a failure to generate sufficient reducing equivalents from core catabolic pathways—is the direct cause of their OXPHOS collapse and bioenergetic deficit. It establishes a direct mechanistic link between the synovial inflammatory microenvironment or ferroptosis stress (which may drive this metabolic rewiring), bioenergetic failure, and the ultimate susceptibility to Tregs functional integrity. Our work also demonstrates that ferroptosis accelerates glycolytic flux—a maladaptive shift that licenses Th17‐like conversion. This metabolic crisis precedes and enables phenotypic dysfunction, explaining why synovial Tregs lose regulatory capacity despite adequate numbers in RA.

Unexpectedly, this study identifies the Tec family kinase TXK as a critical molecular sensor and effector of this ferroptosis‐induced ETC damage. While TXK has known roles in Th1 signaling [18, 19], its specific upregulation in response to ferroptotic stress or direct ETC inhibition in Tregs, and TXK activity is required for the phosphorylation of STAT3 at Tyr705 (not Ser727 as in conventional T cell activation [32, 33]) and PLCγ1 at Tyr783, positioning it as a critical upstream regulator of this signaling node. This signaling axis diverges from classical TCR‐mediated pathways and precisely explains the observed transcriptional shift: downregulation of Treg identity/suppressive genes and upregulation of pro‐inflammatory mediators. PLCγ1 activation likely further amplifies instability via calcium flux and PKC/NFAT signaling [34]. Thus, TXK acts as a metabolic stress switch, converting mitochondrial dysfunction into an inflammatory Treg phenotype. This site‐specific phosphorylation cascade explains how TXK inhibition selectively reverses Th17‐like conversion without compromising Treg survival, revealing a microenvironment‐dependent signaling axis central to Treg dysfunction.

Our findings delineate two synergistic therapeutic avenues for RA: microenvironmental correction through iron chelation and precision targeting of the TXK signaling node. Intra‐articular ceruloplasmin administration effectively reversed synovial iron overload, rescued Treg mitochondrial function, and attenuated experimental arthritis by addressing the root pathological trigger. While this strategy demonstrates broad microenvironmental modulation, its clinical translation necessitates advanced delivery platforms (e.g., stimuli‐responsive nanoparticles) to optimize joint‐specific bioavailability. Conversely, TXK inhibition represents a paradigm‐shifting approach that directly intercepts the core pathogenic cascade—specifically blocking STAT3/PLCγ1 phosphorylation to prevent Treg inflammatory reprogramming while preserving cellular viability, thereby offering precise immune recalibration without systemic immunosuppression. To advance these strategies, future work must prioritize biomarker‐guided patient stratification (synovial Fe^2^^+^/Treg‐NDUFA1 signatures), develop next‐generation intra‐articular delivery systems, explore sequential combination regimens (iron normalization followed by TXK inhibition), and crucially validate TXK pathway druggability in human synovial tissue models.

While our study identifies a novel TXK‐STAT3/PLCγ1 axis critical for Treg dysfunction in RA, several limitations should be acknowledged. First, although genetic silencing confirmed the necessity of TXK, the initial mechanistic discovery relied heavily on a pharmacological inhibitor (Txk‐i), which may have off‐target effects on other Tec‐family kinases (e.g., ITK). Second, although we demonstrate that mtROS acts as a key signaling molecule linking ETC impairment to TXK upregulation, the precise molecular sensors—such as metabolic fluctuations or mitochondrial DAMPs (e.g., mtDNA, ATP, cardiolipin)—remain to be identified, representing a gap in the signal transduction cascade. Third, confirming TXK/STAT3/PLCγ1 axis functionality and inhibitor efficacy in human systems—using *ex vivo* synovium cultures or humanized models—is an indispensable prerequisite for clinical translation [35, 36, 37]. Last, the preliminary evidence of fibroblast/macrophage‐Treg crosstalk modulating ferroptosis susceptibility (Figure S9) necessitates systematic investigation into how stromal cell ferroptosis indirectly impacts Treg fate through altered cytokine milieus or contact‐dependent signaling.

The above limitations delineate clear paths for future investigation: employing more selective TXK inhibitors or conditional knockout models, identifying the upstream TXK inducer(s), and validating the pathway in human Tregs and patient‐derived samples. These investigations will not only deepen our understanding of TXK's role in RA but also help test a broader hypothesis.​ Our findings resonate with the broader “epithelial barrier theory” of chronic inflammatory diseases [38, 39]. This theory posits that exposure to environmental toxins disrupts epithelial barriers, triggering alarmin release, dysbiosis, and chronic type 2 immunity. We propose an extension: in established chronic inflammatory milieus (e.g., the RA synovium), the consequent metabolic and oxidative stress (e.g., iron overload, ferroptosis) may breach intracellular functional barriers within immune cells themselves, such as mitochondrial integrity in Tregs. TXK emerges as a critical intracellular alarm sensor for such stress, converting metabolic catastrophe into a pro‐inflammatory cellular fate. Thus, targeting TXK may represent a strategy to stabilize not only tissue barriers but also the functional barrier of immune regulation. Investigating whether analogous kinase‐mediated stress sensing operates in other immune cells across diseases characterized by barrier dysfunction and immune dysregulation will be a fascinating avenue, potentially unifying mechanisms across diverse chronic inflammatory conditions.

In conclusion, our findings establish the paradigm of microenvironmental stress inducing specific ferroptosis, leading to metabolic dysfunction (mitochondrial ETC damage), which then activates a dedicated TXK‐STAT3/PLCγ1 signaling pathway to reprogram Treg cell fate, has implications beyond RA. Mitochondrial integrity is increasingly recognized as fundamental to Treg stability [27, 28], and TXK or analogous kinases may serve as metabolic stress sensors in other autoimmune or inflammatory conditions characterized by local oxidative stress or metal ion dysregulation (e.g., SLE, inflammatory bowel disease, atherosclerosis). Our work not only provides a mechanistic blueprint for synovial Treg failure in RA but also establishes TXK as a novel therapeutic target for restoring immune tolerance. Targeting this synovium‐specific axis—either by correcting the iron imbalance or directly inhibiting TXK signaling—holds significant promise for developing more effective and potentially tolerance‐inducing therapies for RA.​

## 4 Experimental Section

### 4.1 Mice

Foxp3^RFP^.Il17a^GFP^ mice were developed on a C57BL/6 genetic background through intercrossing the appropriate parental strains. All animal experiments were performed in strict adherence to the guidelines set forth in the approved animal use protocol by the Institutional Animal Care and Use Committee at Shanghai Jiao Tong University School of Medicine (No. 2024–58). Experimental procedures conformed to both institutional and national regulations regarding the care and use of laboratory animals, and all mice utilized in the studies were between 6 and 8 weeks of age to avoid age‐related differences in immune response, consistent with prior experimental arthritis model studies. In this study, both male and female mice were employed, as our prior findings indicated no significant differences between the sexes in the outcomes presented in this manuscript.

### 4.2 Patient Recruitment and Ethics

Samples of synovial tissues were obtained from patients who fulfilled the 2010 ACR/EULAR classification criteria for RA and who underwent arthroplasty at Songjiang Hospital Affiliated to Shanghai Jiao Tong University School of Medicine. Detailed baseline patient information, including levels of C‐reactive protein, rheumatoid factor (RF), anti‐cyclic citrullinated peptide (anti‐CCP) antibody status, erythrocyte sedimentation rate (ESR), duration of the disease, and Disease Activity Score 28 (DAS28), is presented in Table S1. All patient data underwent de‐identification through unique coding, with access restricted to verified researchers via encrypted databases, complying with institutional guidelines. Informed consent was secured from all participants, and the study protocol was approved by the Institutional Review Board of Songjiang Hospital Affiliated to Shanghai Jiao Tong University School of Medicine Ethics Committee (No. 02‐411‐01).

### 4.3 Sample Preparation for 10× Genomics

Initially, the synovial tissues were rinsed with phosphate‐buffered saline (PBS), then cut into small fragments and subjected to enzymatic digestion. Tissue dissociation was performed for 5 min at 37°C in a solution comprising PBS supplemented with 10% fetal bovine serum (FBS), collagenase IV (3 mg/mL), dispase II (1.5 mg/mL), and DNase I (0.1 mg/mL). After three consecutive digestion cycles, the resulting supernatants were collected, combined, and passed through a 70‐µm cell strainer. Subsequently, mononuclear cells were isolated from the synovial membrane tissues using Ficoll–Paque gradient centrifugation (CL5020, CEDARLANE). The number and viability of the isolated cells were assessed using Trypan Blue exclusion staining.

### 4.4 10× Genomics Single‐Cell RNA Sequencing and Data Processing

Single‐cell transcriptomes were captured using the Chromium system (10× Genomics). Libraries were constructed with the Chromium Single Cell 3′ Library & Gel Bead Kit v3 and subsequently sequenced by Gene Denovo Biotechnology Co., Ltd. (Guangzhou, China) on an Illumina NovaSeq 6000 platform. Raw gene expression matrices for individual samples, generated via CellRanger v3.1.0, were integrated in R v3.5.3 using the GRCh38 reference genome for alignment. These integrated data were imported into a Seurat object using the Seurat R package (v3.0.1). This package enabled the assessment of quality control metrics and the filtering of cells. Cells were excluded from analysis if they had more than 20 000 unique molecular identifiers (UMIs), expressed over 4000 genes or fewer than 500 genes, or if more than 10% of UMIs were derived from the mitochondrial genome. To discern variations among different cell types, t‐distributed stochastic neighbor embedding (t‐SNE) was employed using the “RunTSNE” function, with a resolution parameter set to 0.5. Differentially expressed genes were subsequently identified using the Wilcoxon test as implemented in the “FindAllMarkers” function. A gene was considered significantly differentially expressed if it demonstrated an average natural logarithm (fold change) of at least 0.25 and a Bonferroni‐adjusted *p‐*value of less than 0.01.

### 4.5 Collection of Synovial Fluid

RA synovial fluid was aspirated from inflamed knee joints during therapeutic arthrocentesis prior to biologic therapy initiation. All samples were centrifuged (2000 g, 10 min, 4°C) to remove cells/debris, aliquoted, and stored at −80°C within 30 min of collection. CIA Synovial fluid was aspirated from the knee joints of CIA mice at the peak of disease (typically around day 45–50 post‐immunization). The joint cavity was lavaged twice with 5 µL of sterile (PBS. The lavage fluids were pooled together and immediately centrifuged (2000 g, 10 min, 4°C) to remove cells and debris. The cell‐free supernatant was aliquoted and stored at ‐80°C until use. All procedures were approved by the Institutional Animal Care and Use Committee.

### 4.6 Measurement of Iron Level in Synovial Fluid

The total irons and ferrous iron (Fe^2^^+^) concentration in the synovial fluid was determined using a commercial Ferric and Ferrous Ion Assay Kit (S1066S, Beyotime) according to the manufacturer's instructions. The procedure involved incubating samples and standards at 37°C for 30 min after the addition of an iron reducer. After this incubation, iron was introduced, followed by an additional incubation period of 60 min. The absorbance of the resulting solutions was then measured immediately using a colorimetric microplate reader at a wavelength of 593 nm.

### 4.7 Immunofluorescence

Synovial tissue samples were initially fixed in 4% paraformaldehyde for 20 min at room temperature, followed by permeabilization with 0.3% Triton X‐100 for 15 min. To block non‐specific binding, the tissue sections were incubated with 5% bovine serum albumin (BSA) for 1 h. Primary antibodies targeting the proteins of interest were then diluted in 1% BSA at 1:200–1:500 and applied to the sections, which were incubated overnight at 4°C in a humidified chamber. After thorough washing with PBS, the sections were incubated with fluorescently labeled secondary antibodies (1:500–1:2000 dilution in 1% BSA) for 1 h at room temperature, shielded from light. Nuclei were counterstained using 4',6‐diamidino‐2‐phenylindole (DAPI) for 5 min. Finally, the sections were mounted with an antifade medium and visualized under a fluorescence microscope.

### 4.8 Immunohistochemistry

Synovial tissue samples were fixed overnight in 4% paraformaldehyde, then dehydrated and embedded in paraffin. Sections of 4 µm thickness were cut for further analysis. Antigen retrieval was performed by heating the sections in citrate buffer (pH 6) in a microwave for 15 min, followed by cooling to room temperature. To block endogenous peroxidase activity, the sections were treated with 3% hydrogen peroxide for 10 min. Non‐specific binding was prevented by incubating the sections with 5% normal goat serum for 1 h. Primary antibodies targeting the desired proteins were diluted in 1% BSA at 1:200–1:500 and applied to the sections, which were incubated overnight at 4°C in a humidified chamber. After washing with PBS, the sections were incubated with a biotinylated secondary antibody (1:100–1:500 dilution in 1% BSA) for 30 min, followed by a 30‐minute incubation with an avidin‐biotin complex (ABC) reagent. The antigen–antibody complexes were visualized using diaminobenzidine (DAB) as a chromogen, producing a brown precipitate at sites of antigen expression. The sections were then counterstained with hematoxylin, dehydrated, and mounted with a permanent medium. Images were captured using light microscopy.

### 4.9 Murine Model of Inflammatory Arthritis

Freund's incomplete adjuvant (IFA) was prepared by combining heat‐denatured Mycobacterium (3 mg/mL, Chondrex) with bovine type II collagen (C‐II, 4 mg/mL) in equal volumes, resulting in an emulsion with a final C‐II concentration of 3 mg/mL. As previously described [40, 41, 42], DBA‐1J mice were immunized by intradermal injection of 100 µL of the C‐II mixture at the tail base. Thus, a collagen‐induced arthritis (CIA) model was established. Arthritis development was monitored and scored every 2–3 days to evaluate the incidence of the disease. The severity of arthritis in each mouse was individually assessed and scored according to previously established criteria [35, 43, 44]. Individual paw severity was evaluated using a validated 0–4 scoring: 0 = no pathology; 1 = ankle/tarsal edema; 2 = ankle‐tarsal swelling; 3 = ankle‐metatarsal inflammation; 4 = pan–paw involvement/ankylosis. Total arthritis scores (max 16/mouse) represented summed values. Paw swelling thickness was measured every 2–3 days. On day 60, the mice were euthanized via CO_2_ inhalation followed by cervical dislocation. Mice were euthanized if they exhibited >20% weight loss or severe immobility, in accordance with IACUC guidelines.

### 4.10 Isolation of Tregs From Mouse Peripheral Lymphoid Organs

Tregs were isolated from mice in accordance with established protocols, using spleens obtained from healthy Foxp3^rfp^.Il17a^gfp^ mice as we previously described [45, 46]. In summary, CD4+ T cells were enriched through negative selection by incubating the splenocytes with biotin‐conjugated anti‐mouse antibodies specific for CD45R/B220, CD8a, CD11b, CD11c, and CD49b (BioLegend), along with EasySep Streptavidin RapidSpheres (50001, STEMCELL Technologies) and an EasySep magnet (18 000, STEMCELL Technologies). Following this enrichment, Tregs were sorted via fluorescence‐activated cell sorting (FACS) on a FACS Aria II instrument (BD Biosciences), using CD4 Alexa Fluor 700 (clone GK1.5) as a detection marker. Tregs were defined as CD4+FoxP3^rfp^+ cells, with a purity for FoxP3 exceeding 98%.

### 4.11 Quantification of GSH

We quantified cellular glutathione (GSH) concentrations using the GSH‐GSH/GSSG Ratio Detection Assay Kit (Abcam, ab138881) according to the manufacturer's protocol. Briefly, whole‐cell lysates were mixed with the GSH assay reagent to initiate a one‐step fluorometric reaction. After a 60‐minute dark incubation, fluorescence was read at 490/520 nm (Ex/Em). GSH levels were interpolated from a standard curve and subsequently normalized to cell viability data for each experimental group.

### 4.12 Quantification of MDA

Intracellular malondialdehyde (MDA) levels were quantified using the Beyotime Lipid Peroxidation MDA Detection Kit (S0131M), strictly adhering to the provided protocol. Briefly, synovial fluid samples and MDA standards were mixed with a thiobarbituric acid (TBA) solution and incubated at 100°C for 15 min. The resulting MDA‐TBA complex was then quantified colorimetrically by measuring its optical density at 532 nm.

### 4.13 Measurement of Cell Death, Cell Viability, Intracellular Lipid Peroxidation

Cell death was evaluated using SYTOX Green staining (S34860, ThermoFisher Scientific), with subsequent analysis performed by microscopy or flow cytometry. Cell viability was determined using the Cell Titer‐Glo Luminescent Cell Viability Assay, in line with the manufacturer's protocol. ATP levels were measured and normalized to those of control cells to assess viability. Intracellular ROS levels were quantified by staining cells with a 5 µm solution of the C11 Lipid Peroxidation Probe (23290, AAT Bioquest) for 30 min at 37°C, followed by flow cytometric analysis.

### 4.14 Flow Cytometric Analysis for Slc7a11 and Gpx4

To exclude non‐viable cells, the samples were initially stained with Fixable Viability Dye eFluor 780 (catalog number 65‐0865‐14, ThermoFisher Scientific). For the detection of surface Slc7a11, cells were incubated for 30 min at 4°C with the Slc7a11 antibody (catalog number DF12509, Affinity Biosciences) in MACS buffer containing 0.5% BSA and 2 mm EDTA. This was followed by a 30‐minute incubation with Alexa Fluor 647 Donkey anti‐rabbit IgG Antibody (BioLegend) in the same buffer. To perform intracellular staining of Gpx4, cells were first fixed and permeabilized using the eBioscience Foxp3/Transcription Factor Staining Buffer Set (catalog number 00‐5523‐00, eBioscience), as per the manufacturer's instructions. After fixation, the cells were incubated for 30 min at 4°C with the intracellular Gpx4 antibody (catalog number 67763‐1‐Ig, Proteintech) in Perm buffer. Following washing, the samples were resuspended in MACS buffer for analysis. Data were acquired using a CytekTM Northern Lights flow cytometer (Cytek Biosciences) and analyzed using FlowJo software.

### 4.15 Flow Cytometric Analysis for Treg Phenotypic and Functional Genes

For surface staining, cells were incubated with the relevant antibodies in MACS buffer for 30 min at 4°C. Intracellular cytokine analysis required a 5‐hour stimulation using 50 ng/mL phorbol 12‐myristate 13‐acetate (PMA; Sigma P1585), 500 ng/mL ionomycin (Sigma I0634), and protein transport inhibitor Brefeldin A (BioLegend 420601). Stimulated cells were subsequently fixed and permeabilized according to instructions for the Cyto‐Fast Fix/Perm Buffer Set (BioLegend 426803). Flow cytometry data collected on a Cytek Northern Lights platform were processed in FlowJo.

### 4.16 Treg Suppression Assay

Splenic T cells were isolated from wild‐type C57BL/6J mice using nylon wool columns and subsequently labeled with 1 µm Cell Trace carboxyfluorescein diacetate succinimidyl ester (CFSE) (C34554, Thermo Fisher Scientific). The labeled cells were stimulated with an anti‐CD3 antibody at a concentration of 0.05 µg/mL in the presence of antigen‐presenting cells (APCs) that had been treated with mitomycin C, at a ratio of 1:1. Following stimulation, the T cells were cocultured with Tregs for three days. The dilution of CFSE in both CD8+ and CD4+ T cells was analyzed using flow cytometry as we previously described [47, 48].

### 4.17 Protein Digestion and Desalting

Protein aliquots were reduced with 10 mm TCEP (30 min, 37°C) and alkylated with 25 mm CAA (30 min, 37°C). Samples were diluted in 10 mm TEAB to optimal trypsin concentration, then digested overnight at 37°C with sequencing‐grade trypsin (1:50 w/w enzyme: protein). Digestion was terminated by acidification to pH <3 with formic acid. Peptides were desalted using C18 StageTips: conditioned with 100 µL 100% ACN and 100 µL 0.1% FA (centrifugation at 1200 rpm, 3 min). Acidified digests were loaded, washed with 0.1% FA (2 × 100 µL), rinsed with pH 10 H_2_O (100 µL), and eluted with 70% ACN. Eluates were lyophilized and stored at −80°C.

### 4.18 Phosphopeptide Enrichment

Dried peptides were reconstituted in IMAC Binding Buffer (200 µL; 80% ACN/0.1% TFA). Pre‐equilibrated Fe‐NTA magnetic beads were incubated with peptide solution (60 min, RT, rotation). Beads were magnetically immobilized, flow‐through collected, then washed thrice with IMAC Wash Buffer. Phosphopeptides were competitively eluted using sequential buffers: IMAC Elution Buffer 1 (150 µL, pH 10); IMAC Elution Buffer 2 (150 µL, 1% FA/30% ACN). Eluates were pooled, lyophilized, and stored at −80°C prior to LC‐MS/MS analysis. This stoichiometry analysis was performed by Bejing Qinglian Biotech Co., Ltd.

### 4.19 Transmission Electron Microscopy

Tregs were cultured for 24 h, either with or without the inclusion of CIA‐SF, as previously described. After incubation, the cells were promptly harvested and fixed in a solution of 2.5% glutaraldehyde in 0.1 m phosphate buffer (pH 7.4) at 4°C for 24 h to preserve ultrastructural integrity. Following the initial fixation, the samples were washed with phosphate buffer and subsequently post‐fixed in 1% osmium tetroxide for 2 h at room temperature. The tissues were dehydrated through a series of ethanol concentrations ranging from 50% to 100% and then cleared in propylene oxide. The samples were embedded in epoxy resin and polymerized at 60°C for 48 h. Ultrathin sections, measuring 70–90 nm, were prepared using an ultramicrotome and placed onto copper grids. These sections were stained with uranyl acetate and lead citrate to improve contrast. The ultrastructure of the mitochondria was analyzed using a transmission electron microscope operated at an accelerating voltage of 80 kV, and digital imaging was utilized to provide a detailed examination of mitochondrial morphology.

### 4.20 Seahorse Assays Analyzer for Cellular Oxygen Consumption Rate (OCR)

The metabolic activity of the cells was assessed using the Seahorse XF Analyzer (Agilent Technologies), which allowed for real‐time measurement of OCR and extracellular acidification rate (ECAR) [49]. Cells were seeded in XF‐96 cell culture microplates at a density of 2 × 10^5^ cells per well. Prior to the assay, the cells were equilibrated in Seahorse XF assay medium, enriched with optimal concentrations of glucose, pyruvate, and glutamine, at 37°C in a non‐CO_2_ incubator for one hour. A mitochondrial stress test was performed, consisting of four cycles that each included a 3‐minute mixing phase followed by a 3‐minute measurement phase, to evaluate OCR and ECAR both under basal conditions and in response to CIA‐SF, 1 µm Antimycin A (AA, to inhibit mitochondrial complex III, A8674, Sigma Aldrich), 5 µm oligomycin, 1.5 µm of Trifluoromethoxy carbonylcyanide phenylhydrazone (FCCP, C2920, Sigma Aldrich), 5 mm 2‐Deoxy‐*D*‐glucose (2‐DG, to inhibit glucose oxidation, HY‐13966, MCE), and 40 µm etomoxir (to inhibit FAO, HY‐50202, MCE).

### 4.21 Determination of Intracellular ATP

Tregs underwent 24‐hour culture per established methods in either CIA‐SF‐supplemented or control media, with 5 µm AA serving as a positive control for ATP reduction. Cellular ATP levels were assessed using the Perkin Elmer ATPlite Luminescence Assay System (#6016941). Following manufacturer guidelines, post‐incubation lysates were generated via 5‐minute agitation in lysis buffer. Substrate solution was subsequently added prior to 15‐minute dark incubation. Luminescence intensity was quantified on a BioTek Epoch2 microplate reader.

### 4.22 Mitochondrial Membrane Potential Measurements

To evaluate mitochondrial membrane potential, the TMRE (Tetramethylrhodamine ethyl ester)‐Mitochondrial Membrane Potential Assay Probe (115532‐52‐0, AAT Bioquest) was utilized according to the manufacturer's instructions. In brief, Tregs were cultured and activated for 24 h, either with or without CIA‐SF and AA, as previously detailed. AA at a concentration of 10 µm was used as a positive control and added to the cells 10 min before the end of the incubation period. After incubation, TMRE was introduced to the cells, which were allowed to incubate for an additional 20 min at 37°C. Following this treatment, the cells were washed and analyzed by flow cytometry using a Cytek Northern Lights system.

### 4.23 MitoTracker Analysis

Mitochondrial content was evaluated by introducing MitoTracker Red (abs47038879, Absin) into the cell culture medium at a final concentration of 100 nm. The cells were incubated for 30 min at 37°C. After incubation, the cells were rinsed with ice‐cold PBS (17‐516F, LONZA) and subsequently analyzed using flow cytometry on a Cytek Northern Lights system.

### 4.24 Mitochondrial ROS Measurements

The generation of reactive ROS in the mitochondria of Tregs was assessed using the MitoSOX Red Mitochondrial Superoxide Indicator kit (M19992, AbMole), according to the manufacturer's instructions. Briefly, Tregs were incubated for 24 h in the presence or absence of CIA‐SF and AA. Following incubation, the cells were washed to remove the culture medium, and a working solution of MitoSOX reagent was added. The cells were then incubated for 10 min at 37°C. Afterward, the cells were washed again, and fluorescence intensity was measured using flow cytometry on a Cytek Northern Lights system.

### 4.25 Stable Isotope Tracing and Metabolomics Analysis

Tregs were cultured in substrate‐limited medium supplemented with 10 mM [U‐^13^C]‐glucose or 100 µm [U‐^13^C]‐palmitate (complexed to fatty acid‐free BSA) for 4 h. Metabolites were extracted with ice‐cold 80% methanol containing internal standards. After derivatization with methoxyamine hydrochloride and MSTFA, samples were analyzed by gas chromatography‐mass spectrometry (GC‐MS, Agilent 7890A/5977C). Mass isotopomer distributions (MIDs) of TCA cycle intermediates and ^13^CO_2_ production were quantified and corrected for natural isotope abundance using IsoCor software [50].

### 4.26 RNA Sequencing (RNA‐Seq)

RNA isolation from homogenized tissues or cells was carried out with the EZbioscience RNA extraction kit per the manufacturer's protocol. RNA quality and quantity were evaluated using a NanoDrop spectrophotometer and an Agilent 2100 Bioanalyzer. For RNA sequencing, we constructed libraries with the NEBNext Ultra DNA Library Prep Kit for Illumina (v6.0–2/18) as directed. Briefly, poly(A) selection isolated mRNA, which was then fragmented and reverse‐transcribed into cDNA. The cDNA underwent end repair and adaptor ligation. Post‐PCR amplification, AMPure XP beads purified the libraries, which were then assessed for integrity and size distribution on the Agilent 2100 Bioanalyzer. The Illumina NextSeq 500 system sequenced the libraries to generate 150 bp paired‐end reads.

The raw sequencing data were analyzed through a standard bioinformatics pipeline, which included trimming adaptor sequences and low‐quality bases using Trimmomatic. Alignment to the reference genome (GRCh38/hg38) was conducted with HISAT2. Gene expression quantification was performed with featureCounts, followed by normalization via the Trimmed Mean of M‐values (TMM) method in edgeR. For differential expression analysis, we employed DESeq2, considering genes with a false discovery rate (FDR) below 0.05 as statistically significant. To interpret the biological implications of the differentially expressed genes, functional enrichment analysis was conducted using Gene Set Enrichment Analysis (GSEA). The results were visualized and interpreted within the R programming environment to elucidate the biological pathways and processes involved under the experimental conditions.

### 4.27 Untargeted Metabolomics Analysis

Cell lysates were first homogenized in a cold methanol/water solution (4:1, v/v) and centrifuged at 14 000 g for 10 min at 4°C to promote protein precipitation. The supernatant was then collected, evaporated under a nitrogen stream, and reconstituted in a 50:50 mixture of acetonitrile and water. To ensure quality control, pooled QC samples were created by combining equal volumes from each individual sample. Metabolite separation and detection were carried out using a high‐resolution ultra‐performance liquid chromatography (UPLC) system coupled with a quadrupole time‐of‐flight mass spectrometer (Q‐TOF MS) (Bruker). Chromatographic separation was achieved with a C18 column maintained at 40°C, utilizing a gradient elution with water and acetonitrile, each containing 0.1% formic acid. The mass spectrometer was operated in both positive and negative electrospray ionization (ESI) modes to maximize metabolite detection. Data acquisition covered a mass range of 50–1000 m/z with a resolution of 30 000, and data analysis was performed using Compound Builder software for peak detection, alignment, and normalization.

Metabolite identification was carried out by comparing accurate mass, retention time, and MS/MS fragmentation patterns to those in the METLIN reference database. Unidentified features were annotated based on their mass‐to‐charge ratios and retention times. To identify metabolites that exhibited significant changes across experimental groups, multivariate statistical methods, including principal component analysis (PCA) and orthogonal partial least squares discriminant analysis (OPLS‐DA), were performed using MetaboAnalyst. Additionally, pathway enrichment analysis was conducted to explore the biological relevance of the observed changes.

### 4.28 Treatment of CIA Mice With Ceruloplasmin (Cp)​

To investigate the therapeutic effect of Cp on arthritis progression, the CIA model was established in DBA/1J mice. On day 15 post‐immunization, when mice began to exhibit clinical signs of arthritis, they were randomly allocated into treatment groups. The Cp‐treated group received intra‐articular injections of Cp (5 mg/kg), while the control CIA group received an equal volume of PBS. This treatment regimen was initiated on day 15 and repeated weekly until day 50 post‐immunization. The incidence of arthritis was monitored daily, and clinical arthritis severity scores were recorded in a blinded manner from day 15 to day 60 post‐immunization. At the experimental endpoint (day 60), serum and synovial fluid were collected, after which the mice were euthanized for the harvesting of hind limb joints and spleen tissue.

A comprehensive analysis was performed to evaluate the therapeutic effects of Cp. The collected joint tissues were fixed, decalcified, paraffin‐embedded, and sectioned for histological analysis. Tissue sections were stained with hematoxylin and eosin (H&E), and histological scoring for synovial hyperplasia and inflammation was performed by an observer blinded to the experimental groups. Cartilage matrix assessment was determined by toluidine blue (TB) staining. Bone erosion in the paws was further quantified using micro‐computed tomography (micro‐CT) imaging. The levels of key inflammatory cytokines, including Tnf‐α, Il‐1β, Il‐6, and Il‐17a, in the serum were measured by enzyme‐linked immunosorbent assay (ELISA). Furthermore, the total irons concentration in the synovial fluid was determined using a commercial iron assay kit. The correlation between synovial iron concentration and clinical arthritis scores was analyzed using linear regression.

### 4.29 Adoptive Treg Transfer for CIA Model and Analysis of Ferroptosis​

To specifically investigate whether Cp protects against arthritis by modulating ferroptosis in Treg cells, an adoptive Treg cell transfer experiment was conducted. Treg cells were isolated from the spleens of B6.Foxp3^RFP^.Il17a^GFP^ double‐reporter mice by sorting for Foxp3‐RFP^+^ cells using flow cytometry. On day 30 post‐immunization, CIA mice were divided into groups. One group received an intravenous injection of the sorted Foxp3‐RFP^+^ Treg cells (approximately 2 × 10^6^ cells per mouse) followed by an intra‐articular injection of Cp (5 mg/kg). The control group received the same number of Treg cells along with an intra‐articular injection of an equal volume of vehicle. All recipient mice were euthanized 5 days after Treg transfer (day 35 post‐immunization). The exogenously transferred Treg cells were sorted through RFP reporter fluorescence from the splenic tissue of the recipient mice for subsequent analysis.

A series of assays related to ferroptosis was performed on the retrieved Treg cells. Lipid ROS production was assessed using the C11‐BODIPY 581/591 probe and flow cytometry. The levels of MDA and GSH were measured using commercial biochemical assay kits. Mitochondrial integrity and reactive oxygen species were evaluated by staining cells with MitoTracker Red dye and MitoSOX Red Mitochondrial Superoxide Indicator. Finally, to assess the stability of Treg cells, the conversion of transferred Treg cells into a pathogenic Th17‐like phenotype was analyzed by flow cytometry. Utilizing the Il17a‐GFP reporter, the percentage of Foxp3‐RFP^+^ Treg cells that had converted to express GFP (indicative of Il‐17a production) was quantified among the re‐isolated cells from the spleen.

### 4.30 Statistics Analysis

Data normality was assessed via Shapiro–Wilk tests. Non‐parametric tests (Mann–Whitney U) were used for non‐normal distributions. Unless stated otherwise, data are presented as mean ± standard deviation (SD). To compare means between two groups, a two‐tailed Student's *t*‐test was utilized. For assessing differences among multiple groups, a one‐way ANOVA with Tukey's post‐hoc was performed for each individual comparison. Statistical analyses were carried out using GraphPad Prism version 9.3 (GraphPad Prism Software). A *p*‐value of less than 0.05 was considered statistically significant.

## Author Contributions

JRC and XG performed the experiment and analyzed the data; JRC and YTJ wrote the manuscript; JRC, XG, XYS, ZJ, RZL, JLD, YC, YDX, CZ, XLF and LZ and helped in data collection; YTJ, FTZ, ZHZ and CXS helped in the collection of synovial tissues and synovial fluid; KXX helped in scRNA‐seq data analysis; LYW and HMW helped in manuscript revision; NO helped in manuscript editing; SGZ conceptualized the research, designed experiments, analyzed data and finalized the manuscript for submission.

## Ethics Statement

All patients’ informed consent was obtained. The study protocol and the use of the material were approved by the School of Cell and Gene Therapy at the Shanghai Jiao Tong University School of Medicine in China (No. 02‐411‐01).

## Conflicts of Interest

The authors declare that they have no competing interests.

## Supporting information




**Supporting File 1**: advs75484‐sup‐0001‐SuppMat.docx.

**Supporting File 2**: advs75484‐sup‐0002‐Raw data.pptx.

## Data Availability

The data that support the findings of this study are available from the corresponding author upon reasonable request.;
