# Supplementary material for: Disruption of Treg Homeostasis in Rheumatoid Arthritis via Ferroptosis‐Mediated ETC Collapse and TXK‐STAT3/PLCγ1 Activation
Source: Adv Sci (Weinh). 2026 May 19;13(41):e20519. doi: 10.1002/advs.202520519 (PMC13335581; doi:10.1002/advs.202520519)
Supplement: Supplementary file 1 — Supporting File 1: advs75484‐sup‐0001‐SuppMat.docx. [file ADVS-13-e20519-s001.docx]

**Supplementary Materials**

**​​ Disruption of Treg Homeostasis in Rheumatoid Arthritis via Ferroptosis-Mediated ETC Collapse and TXK-STAT3/PLCγ1 Activation**

*Jingrong Chen^1^**^,2,3*^, Xiao Guan^1,2*^, Kexu Xiong^4,5*^, Xiaoyi Shi^6^, Luyao Wu^7^, Chuanxin Su^1,2^, Jun Zhao^1,2^, Rongzhen Liang^1,2^, Huimin Wang, Junlong Dang^1,2^, Ye Chen^1,2^, Yiding Xiong^1,2^, Chi Zhou^1,2^, Futao Zhao^8^, Zhenhang Zhu^8^, Xiaoli Fan^1^, Li Zhou^9^, Nancy Olsen^10^, Yutong Jiang^11^ and Song Guo Zheng^1,2,3#^*

1. Division of Rheumatology, Department of Medicine, Songjiang Research Institute, Songjiang Hospital Affiliated to Shanghai Jiao Tong University School of Medicine, Shanghai, 201600, China

2. Department of Immunology, School of Cell and Gene Therapy, Songjiang Research Institute, Songjiang Hospital Affiliated to Shanghai Jiao Tong University School of Medicine, Shanghai, 201600, China

3. The State Key Laboratory of Innovative Immunotherapy at the Shanghai Jiao Tong University School of Medicine, Shanghai, 201600, China

4. Shenzhen Key Laboratory of Viral Oncology, The Clinical Innovation & Research Center (CIRC), Shenzhen Hospital, Southern Medical University, Shenzhen, 518054, China

5. Shenzhen Qianhai Shekou Free Trade Zone Hospital, Shenzhen, 518054, China

6. Department of Organ Transplantation, The Affiliated Guangdong Second Provincial General Hospital of Jinan University, Guangzhou, 510317, China

7. Department of Cardiology, Songjiang Research Institute, Songjiang Hospital Affiliated to Shanghai Jiao Tong University School of Medicine, Shanghai, 201600, China

8. Department of Rheumatology, Ninth People's Hospital, Shanghai Jiao Tong University School of Medicine, Shanghai, 200011, China

9. Division of Oncology, Department of Medicine, Songjiang Hospital Affiliated to Shanghai Jiao Tong University School of Medicine, Shanghai, 201600, China

10. Division of Rheumatology, Department of Internal Medicine, Penn State Hershey Medical Center, Hershey, PA, USA

11. Division of Rheumatology, Department of Internal Medicine, The Third Affiliated Hospital of Sun Yat-sen University, Guangzhou, 510630, China

***These authors contributed equally to this work.**

**#Address correspondence via email to**

Song Guo Zheng. 768 Zhongshan Middle Road, Shanghai, 201600, China. [Song.Zheng@shsmu.edu.cn](mailto:Song.Zheng@shsmu.edu.cn)

**Supplementary results**

**
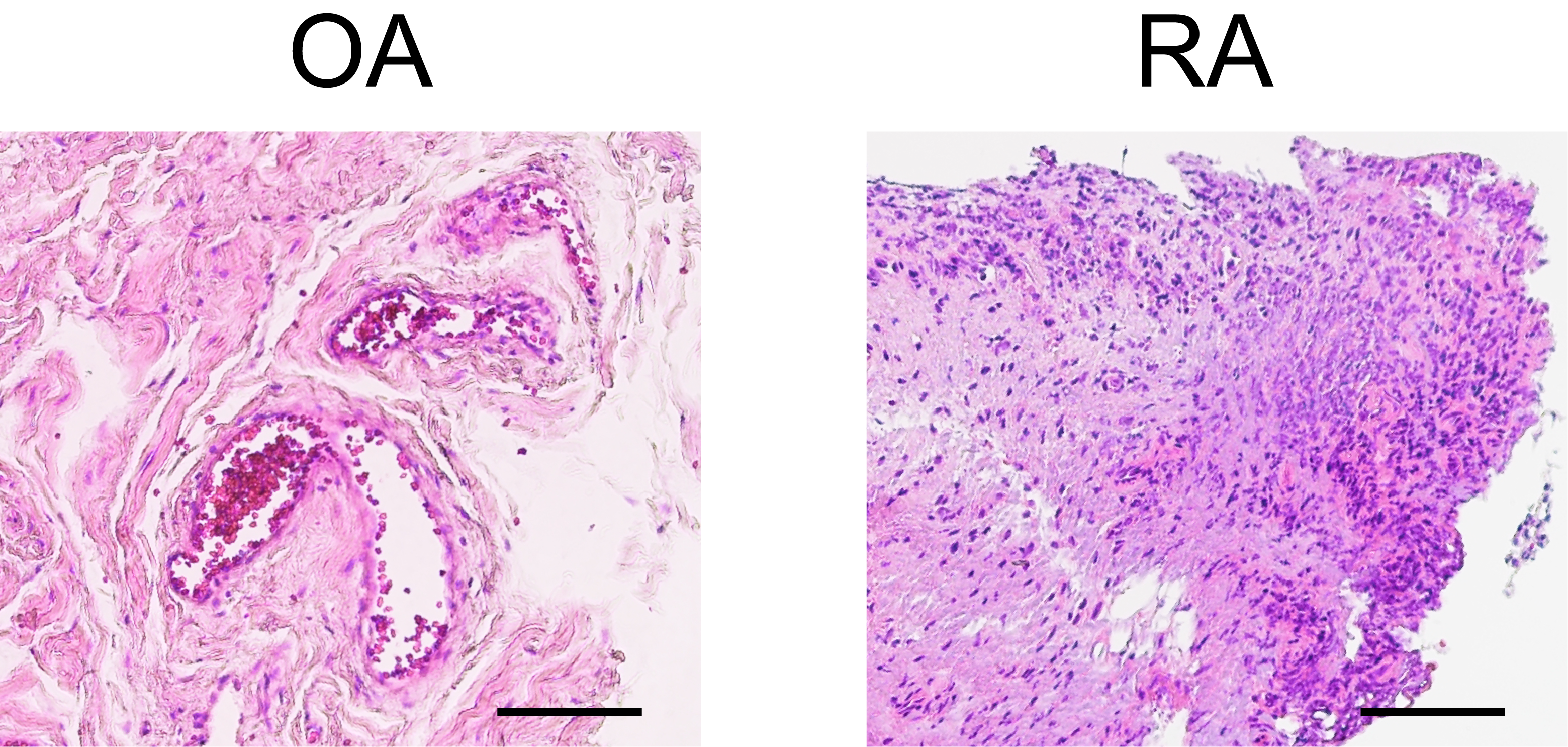
**

**Figure S1.** Representative images from H&E staining of synovial tissues from osteoarthritis (OA) and rheumatoid arthritis (RA) patients. Scale bars, 200 μm.

**
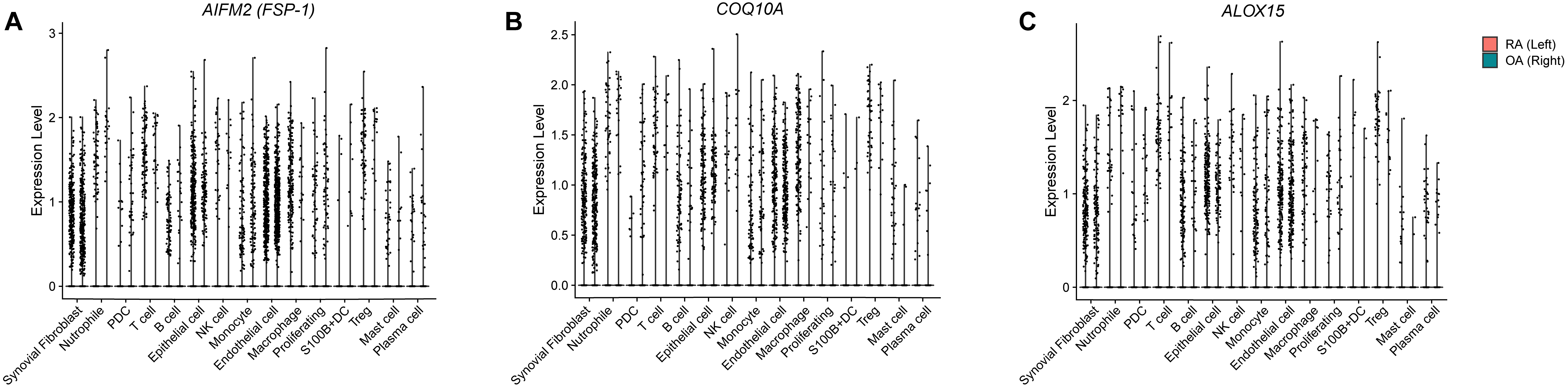
**

**Figure S2. Analysis of AIFM2, COQ10A and ALOX15 expression in synovial tissues.** (**A-C**)​ Violin plot comparing the expression level of AIFM2, COQ10A and ALOX15 mRNA between Tregs and other indicated synovial cell types based on scRNA-seq data.

**
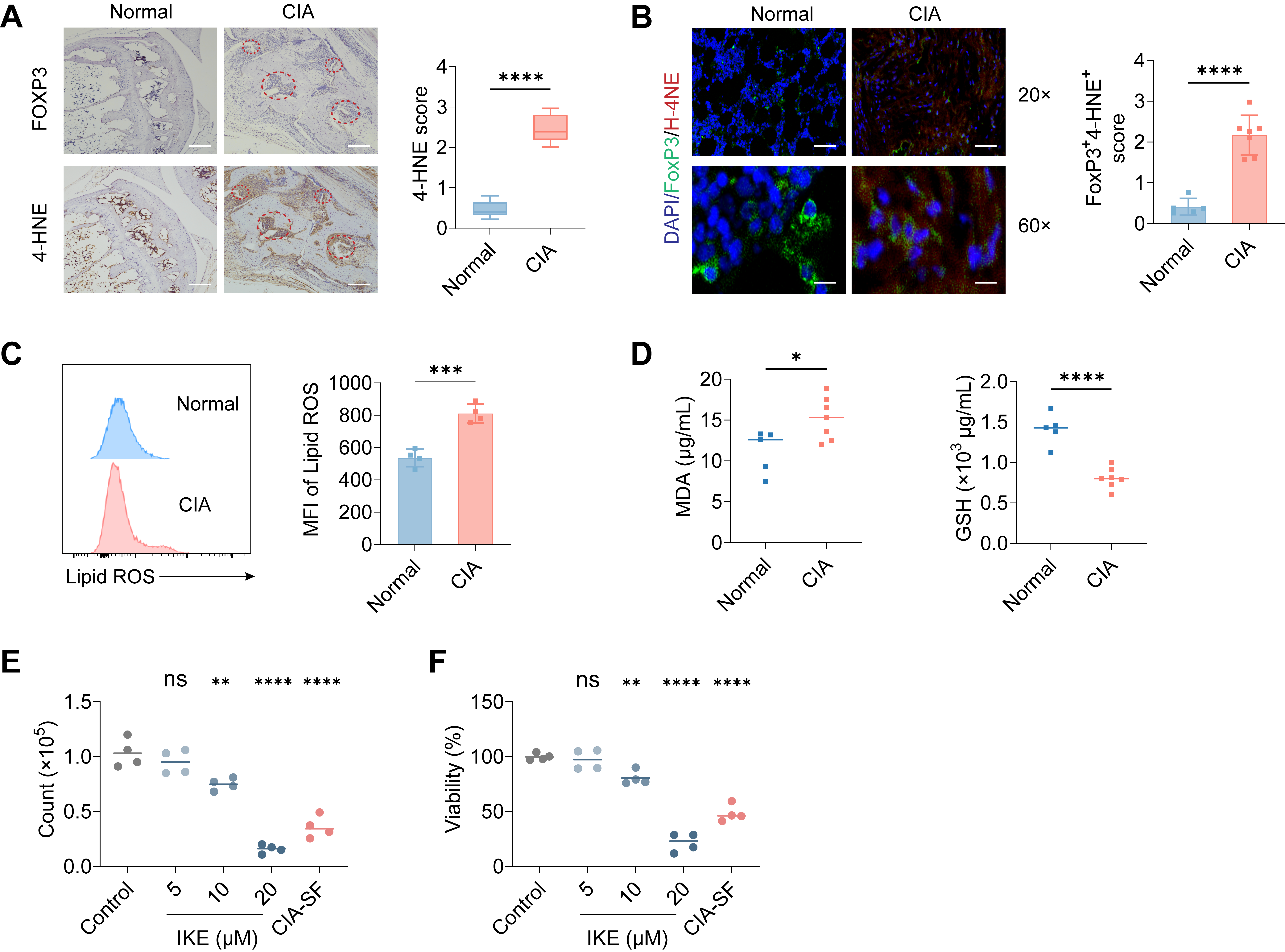
**

**Figure S3. Characterization of ferroptosis in Tregs from CIA mice. (A)** Representative images of immunohistochemistry staining of FoxP3 and 4-HNE in joint from CIA mice. Scale bars, 500 μm. **(B)** Representative images of immunofluorescence staining of FoxP3 and 4-HNE in joint from CIA mice. Scale bars, 200 μm, ×20; Scale bars, 66.7 μm, ×60. **(C)** Assessment of lipid ROS production in Tregs from CIA mice. **(D)** Measurement of lipid peroxide MDA and antioxidant GSH levels in Tregs from CIA mice. **(E)**​​ Representative images of Trypan Blue staining assessing Treg viability following treatment. ​​**(F)**​​ Quantification of relative Treg cell viability. A-D, Data are mean ± SD, n = 5-8 mice. E-F, Data are shown as the means ± SD from one of three independent experiments. *, *p* < 0.05; **, *p* < 0.01; ***, *p* < 0.001; ****, *p* < 0.0001.

**
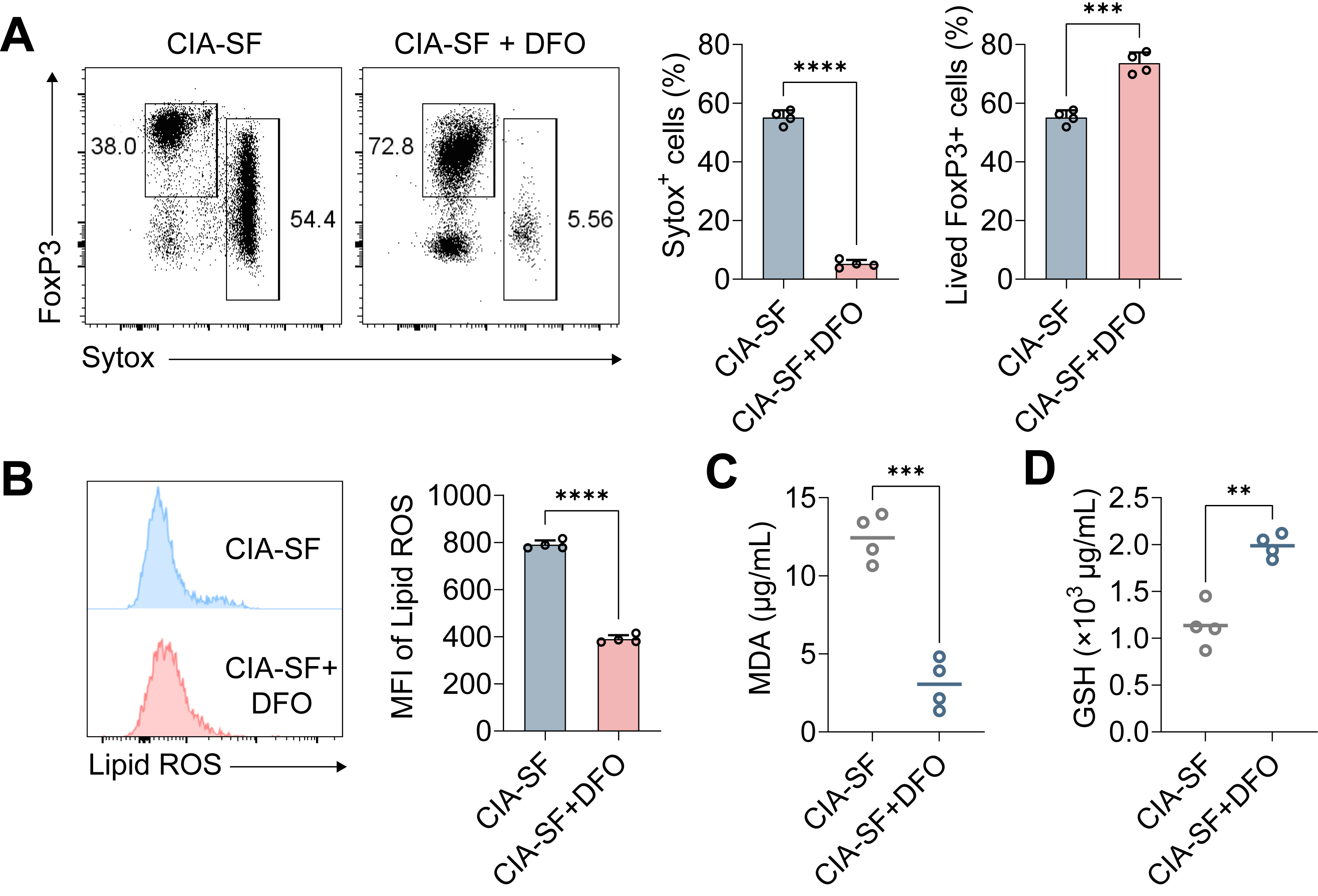
**

**Figure S4. Effects of iron chelator DFO on CIA-SF induced ferroptosis in murine Tregs.** Tregs isolated from C57BL/6J mice were treated with 2% (v/v) CIA-SF in the presence or absence of DFO. **(A)** Quantification of Treg cell death assessed by SYTOX Green staining. **(B)** Measurement of intracellular lipid reactive oxygen species (lipid ROS) production using the C11-BODIPY581/591 probe, analyzed by flow cytometry. **(C)** Assessment the level of MDA. **(D)** Assessment the level of GSH. Data are presented as mean ± SD. **, *p* < 0.01; ***, *p* < 0.001; ****, *p* < 0.0001.

**
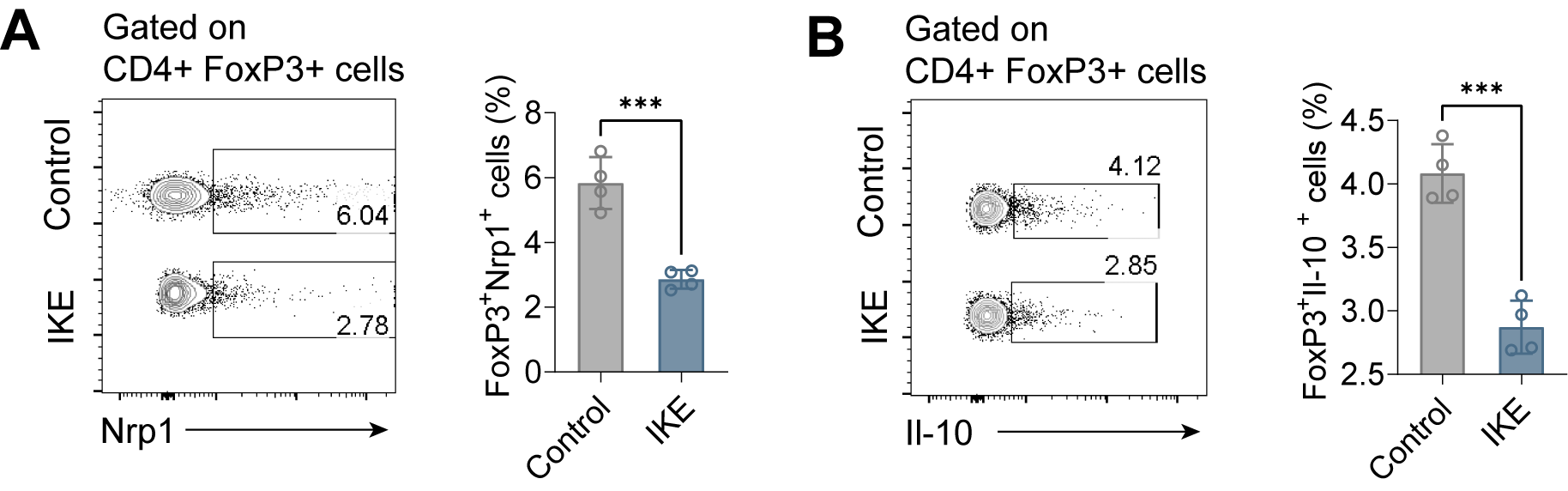
**

**Figure S5.** **Low dose of IKE induces alterations in the phenotype and functionality of Tregs. (A, B)** Purified mouse Tregs were exposed to low dose of IKE or culture medium alone for 48 hours, cells were harvested and analyzed for the expression of Nrp1 and Il10 using flow cytometry. Data are shown as the means ± SD from one of three independent experiments. ***, *p* < 0.001.

**
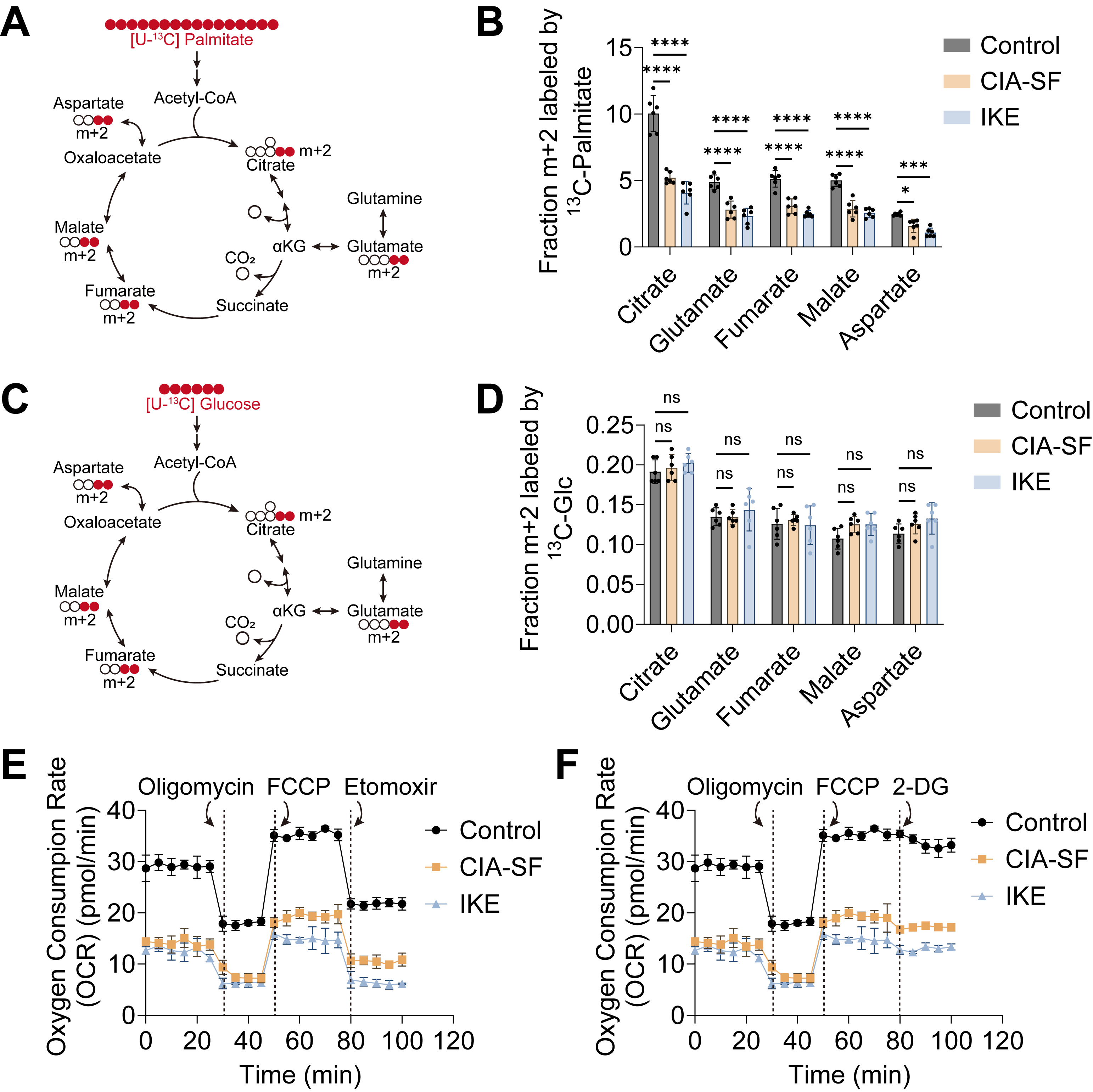
**

**Figure S6. Metabolic analysis of Tregs under ferroptotic stress.** (**A**)​ Schematic of the experimental setup for fatty acid oxidation (FAO) flux analysis using [U-¹³C]-palmitate tracing. (**B**)​ Mass isotopomer distribution (MID) of ¹³C in TCA cycle intermediates (citrate, α-ketoglutarate, succinate, malate) from [U-¹³C]-palmitate in Tregs treated with or without CIA-SF (2% v/v) or IKE (5 µM) for 24 hours. (**C**)​ Schematic of the experimental setup for glucose oxidation flux analysis using [U-¹³C]-glucose tracing. (**D**)​ MID of ¹³C in TCA cycle intermediates from [U-¹³C]-glucose in Tregs treated as indicated in (B). (**E, F**)​ Substrate dependency of mitochondrial respiration. The OCR of Tregs (treated as in B) was measured in real-time using a Seahorse XF Analyzer. Arrows indicate the sequential addition of the oligomycin(5μM), FCCP (1.5μM), etomoxir (40 µM) and 2-DG (5mM).

**
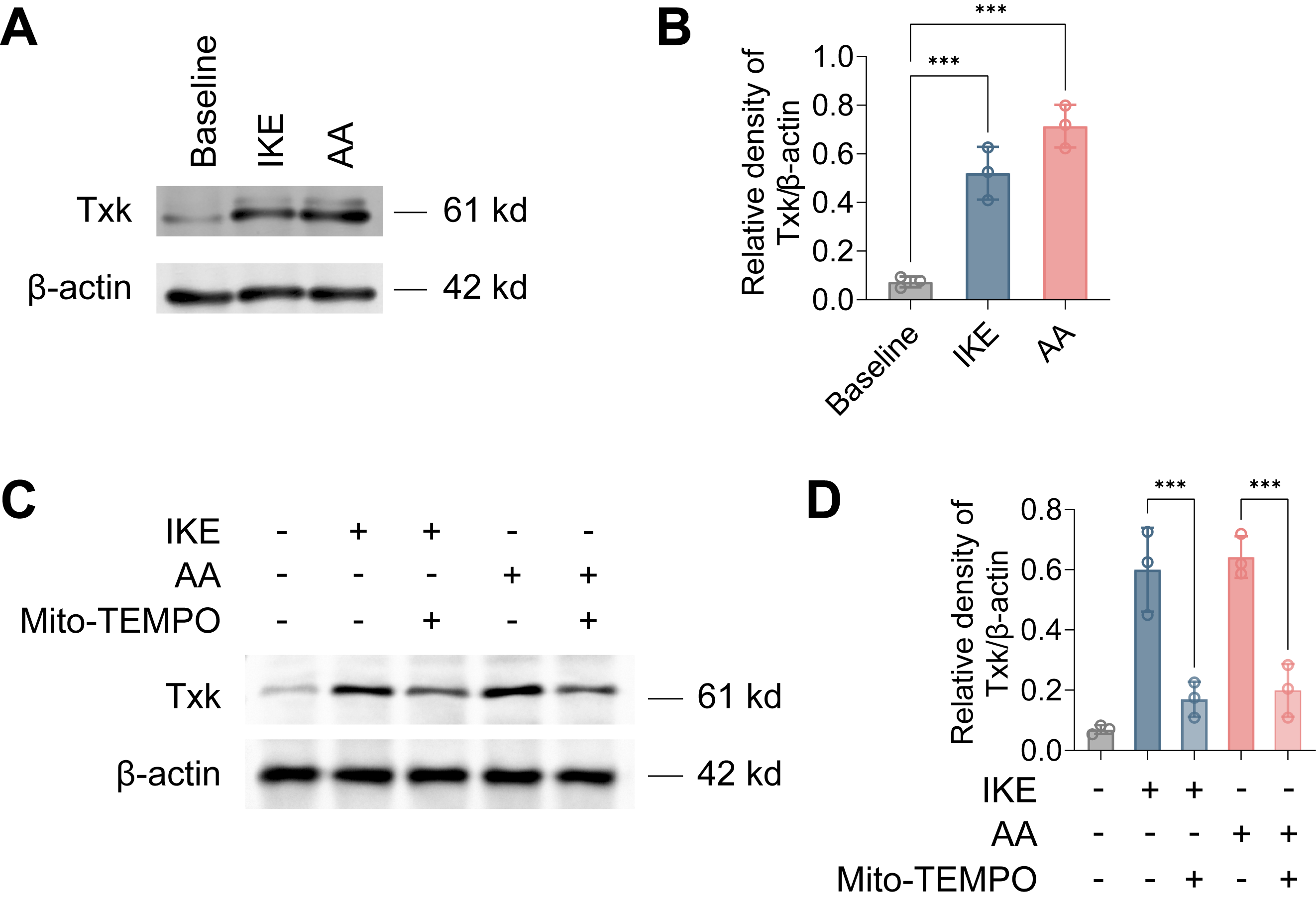
**

**Figure S7. Ferroptotic stress or direct ETC inhibition induces TXK expression. (A, B)** Tregs were treated with IKE (5 μM) or Antimycin A (10 μM) for 24 hours, and relative protein expression levels of Txk were measured. **(C, D)** Quantification of TXK protein expression in Tregs treated with IKE (5 µM) or Antimycin A (10 μM) in the presence or absence of the mitochondrial-targeted antioxidant Mito-TEMPO (100 µM) for 24 hours. Data are shown as the means ± SD from one of three independent experiments. ***, *p* < 0.001.

**
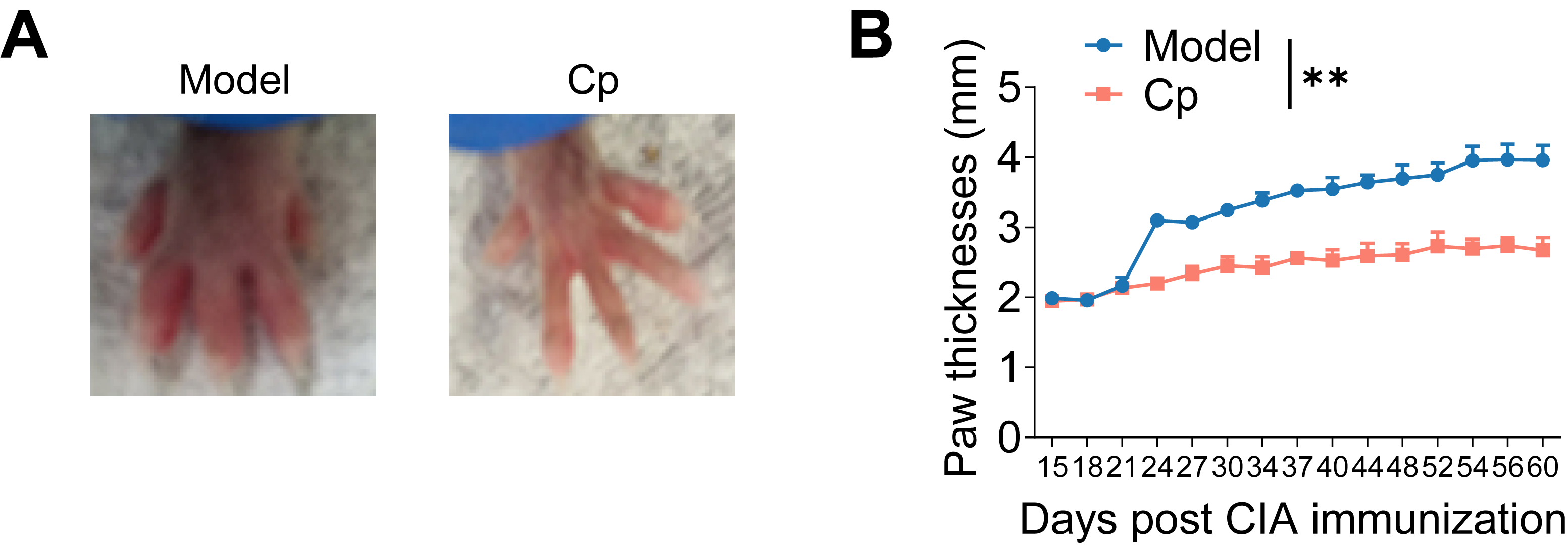
**

**Figure S8. Cp attenuates arthritis progression in CIA mice. (A)** The representative images of gross appearance of swollen hind paws on day 60 after immunization. **(B)** The paw thicknesses of swollen hind paws were monitored from day 15 to day 60 after immunization. Data are mean ± SD, n = 5-8 mice. **, *p* < 0.01.

**
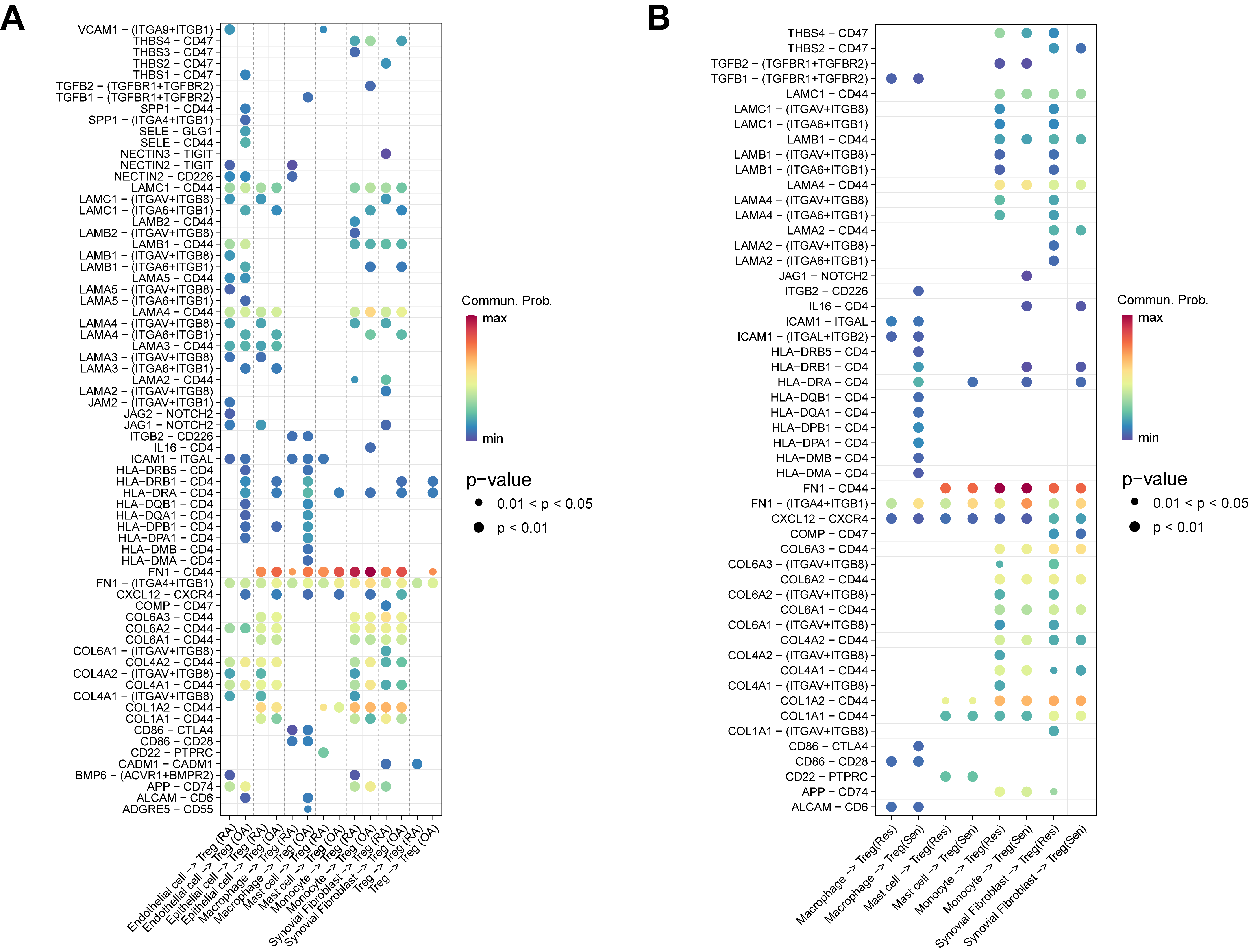
**

**Figure S9. Cell communication network analysis by scRNA-seq. (A)** Interaction strength between cell populations derived from RA and OA synovial tissues and Treg cell-related responses. **(B)** Interaction strength between cell populations derived from RA and OA synovial tissues and ferroptosis-resistant and ferroptosis-sensitive Treg subpopulations.

| **Patient ID** | **Age (years)** | **Gender** | **CRP**  **(mg/dL)** | **ESR**  **(mm/h)** | **WBC**  **(10^9^/L)** | **Lymphocyte**  **(10^9^/L)** | **Monocyte (10^9^/L)** |
| --- | --- | --- | --- | --- | --- | --- | --- |
| OA1 | 58 | Male | 0.27 | 39 | 7.5 | 0.31 | 0.074 |
| OA2 | 62 | Male | 0.19 | 17 | 5.9 | 0.42 | 0.062 |
| OA3 | 65 | Female | 0.43 | 29 | 6.2 | 0.29 | 0.059 |
| RA1 | 67 | Female | 25.8 | 42 | 5.6 | 0.35 | 0.084 |
| RA2 | 64 | Male | 34.9 | 28 | 6.7 | 0.42 | 0.074 |
| RA3 | 55 | Female | 22.7 | 39 | 8.4 | 0.40 | 0.094 |
| RA4 | 61 | Female | 28.4 | 31 | 4.8 | 0.45 | 0.084 |
| RA5 | 67 | Female | 17.9 | 33 | 7.4 | 0.43 | 0.094 |

**Table S1. Clinical information of RA and OA patients for synovial tissue samples for scRNA-seq.** C-reactive protein (CRP), Erythrocyte sedimentation rate (ESR), White Blood Cell (WBC) are shown.

| **Clinical Information** | **RA** | **OA** |
| --- | --- | --- |
| **Number (M/F)** | 10 (3/7) | 10 (6/4) |
| **Age mean, (range)** | 55.5 (50-67) | NA |
| **ACPA (pos/neg)** | 10/1 | NA |
| **RF (pos/neg)** | 9/1 | NA |
| **CRP (mg/dL), mean ± SD** | 22.8 ± 8.5 | 0.25 ± 0.09 |
| **ESR (mm/h), mean ± SD** | 32.6 ± 10.6 | 25.5 ± 9.5 |
| **DAS 28, mean ± SD** | 5.2 ± 2.1 | NA |
| **SJC28, mean ± SD** | 4.3 ± 3.8 | NA |

**Table S2. Clinical information of RA patients for synovial tissue samples included in this study.** Antibodies to citrullinated protein antigen (ACPA), Rheumatoid Factors (RF), C-reactive protein (CRP), Erythrocyte Sedimentation Rate (ESR), Disease Activity Score-28(DAS28) and swollen 28-joint count (SJC28) are shown.
