# Supplementary material for: Disruption of Treg Homeostasis in Rheumatoid Arthritis via Ferroptosis‐Mediated ETC Collapse and TXK‐STAT3/PLCγ1 Activation
Source: Adv Sci (Weinh). 2026 May 19;13(41):e20519. doi: 10.1002/advs.202520519 (PMC13335581; doi:10.1002/advs.202520519)
Supplement: Supplementary file 2 — Supporting File 2: advs75484‐sup‐0002‐Raw data.pptx. [file ADVS-13-e20519-s002.pptx]

## Slide 1
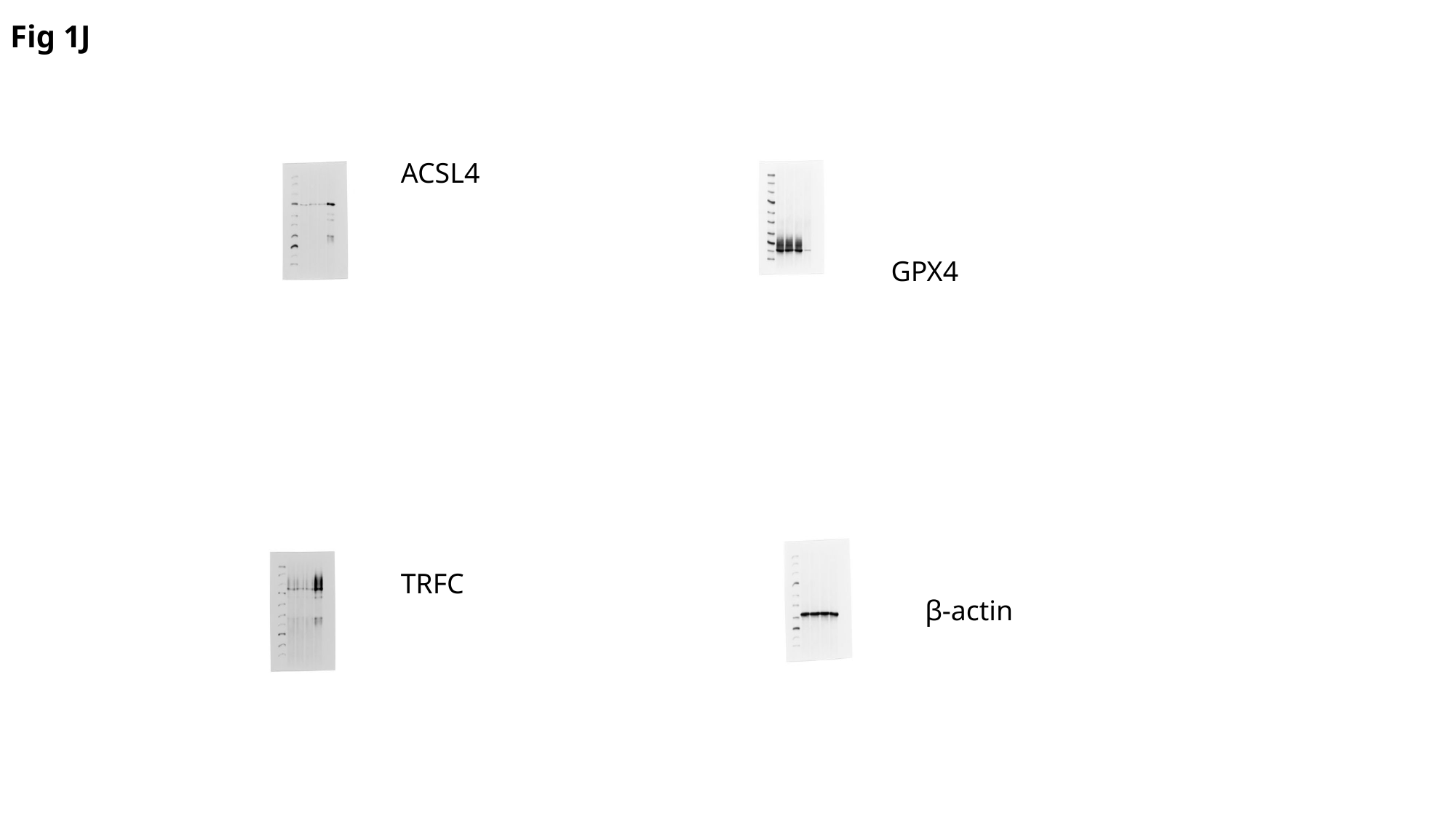

Fig 1J
ACSL4
GPX4
TRFC
β-actin

## Slide 2
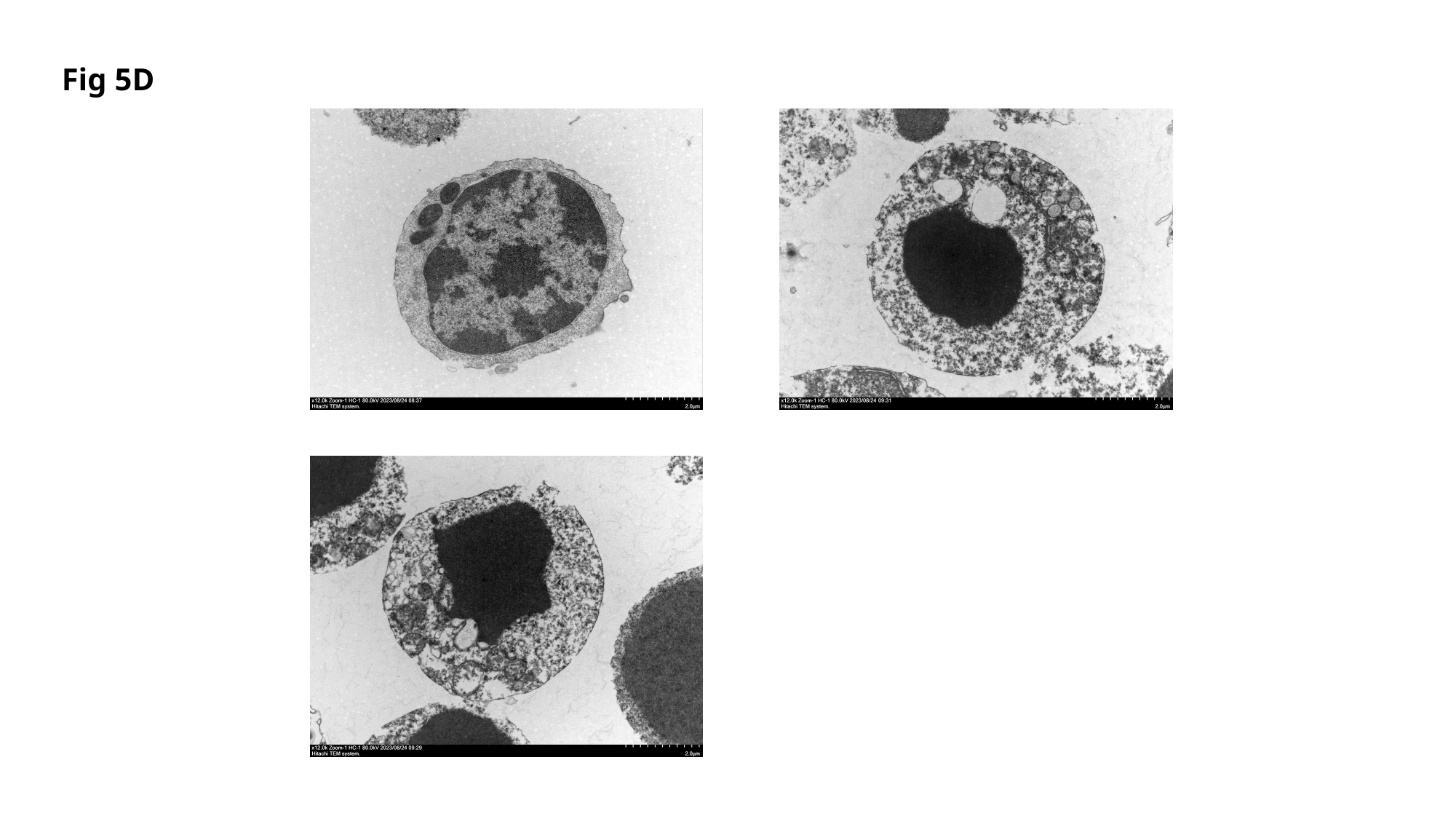

Fig 5D

## Slide 3
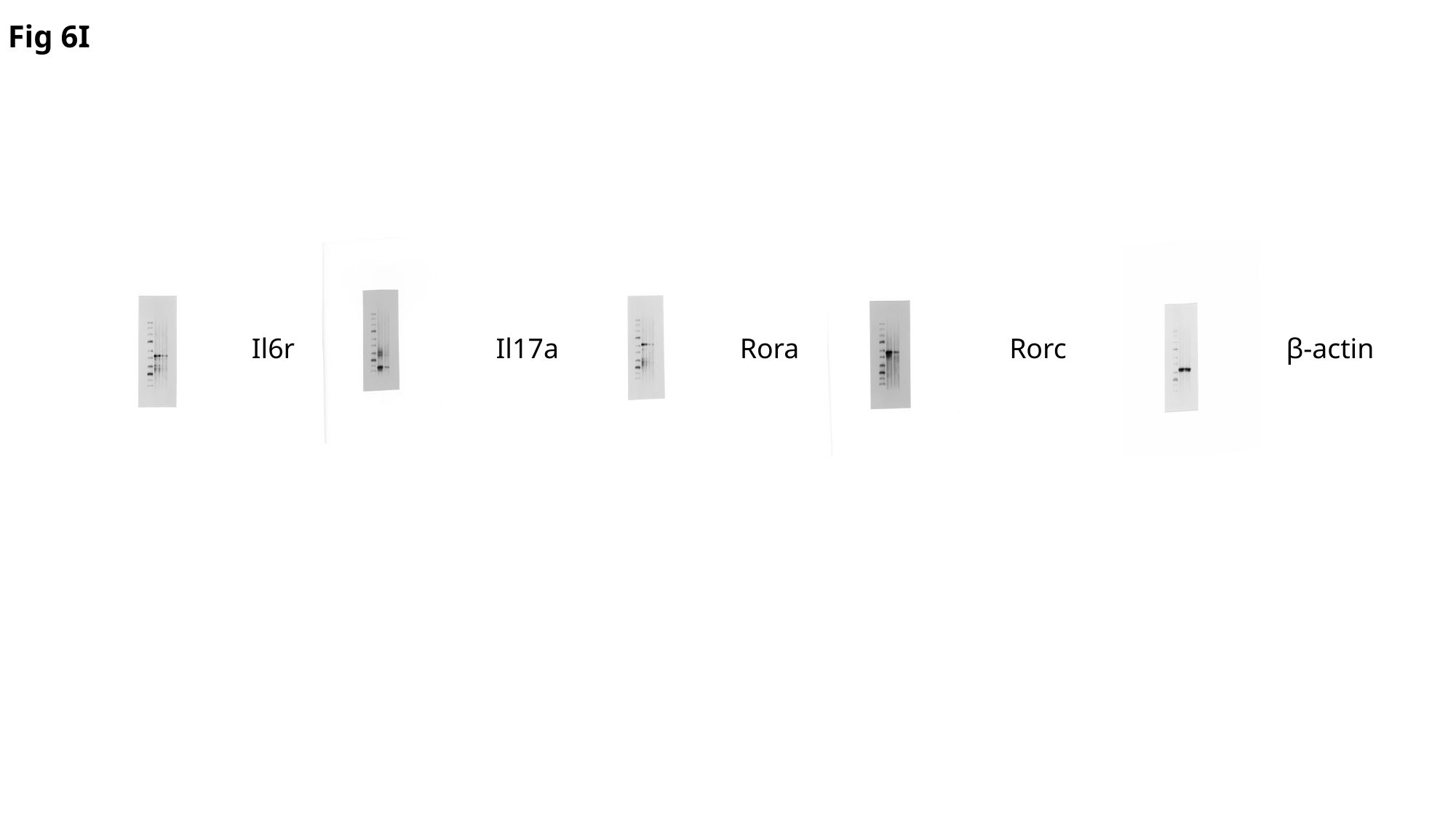

Fig 6I
Il6r
Il17a
Rora
Rorc
β-actin

## Slide 4
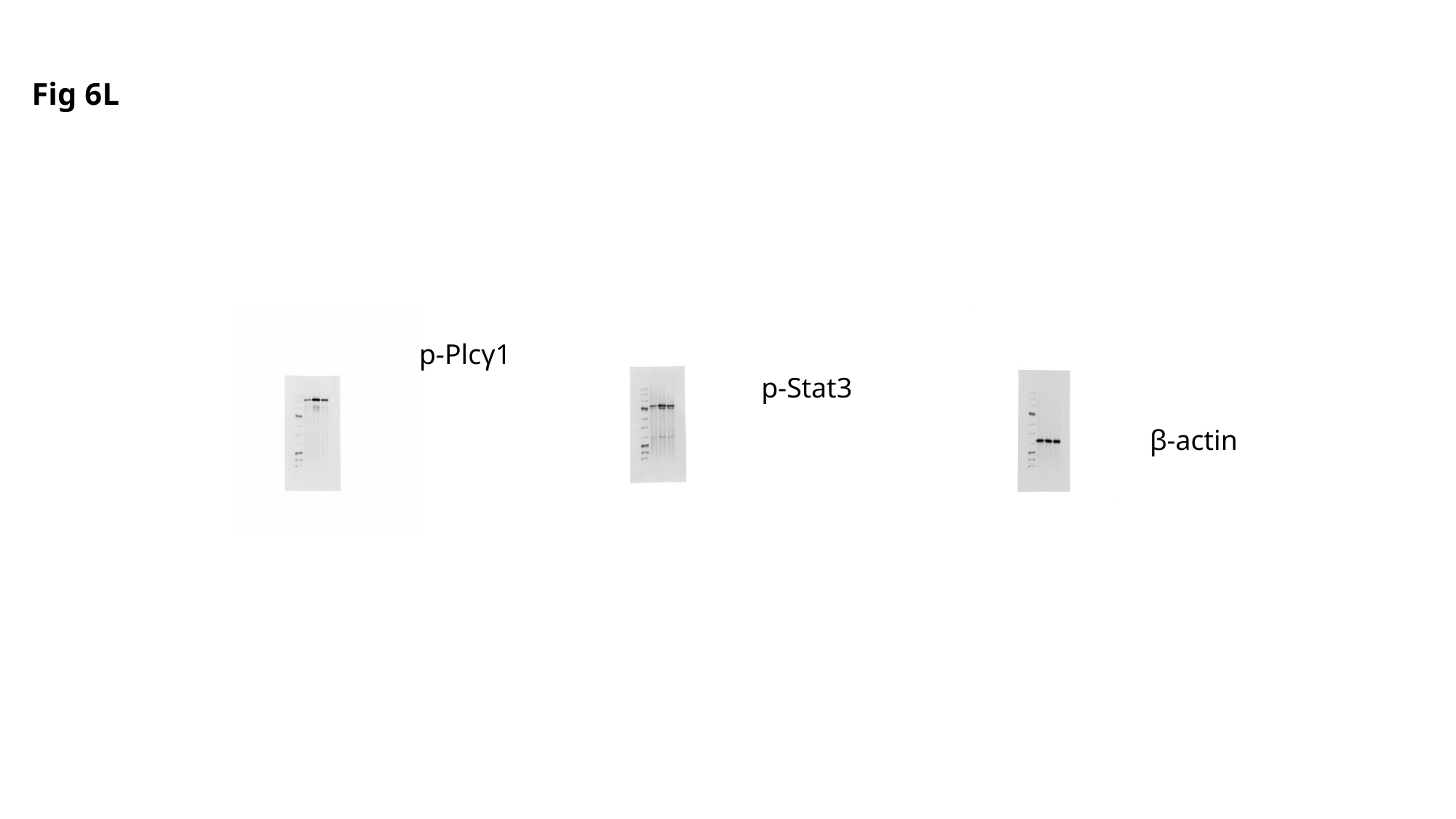

Fig 6L
p-Plcγ1
p-Stat3
β-actin

## Slide 5
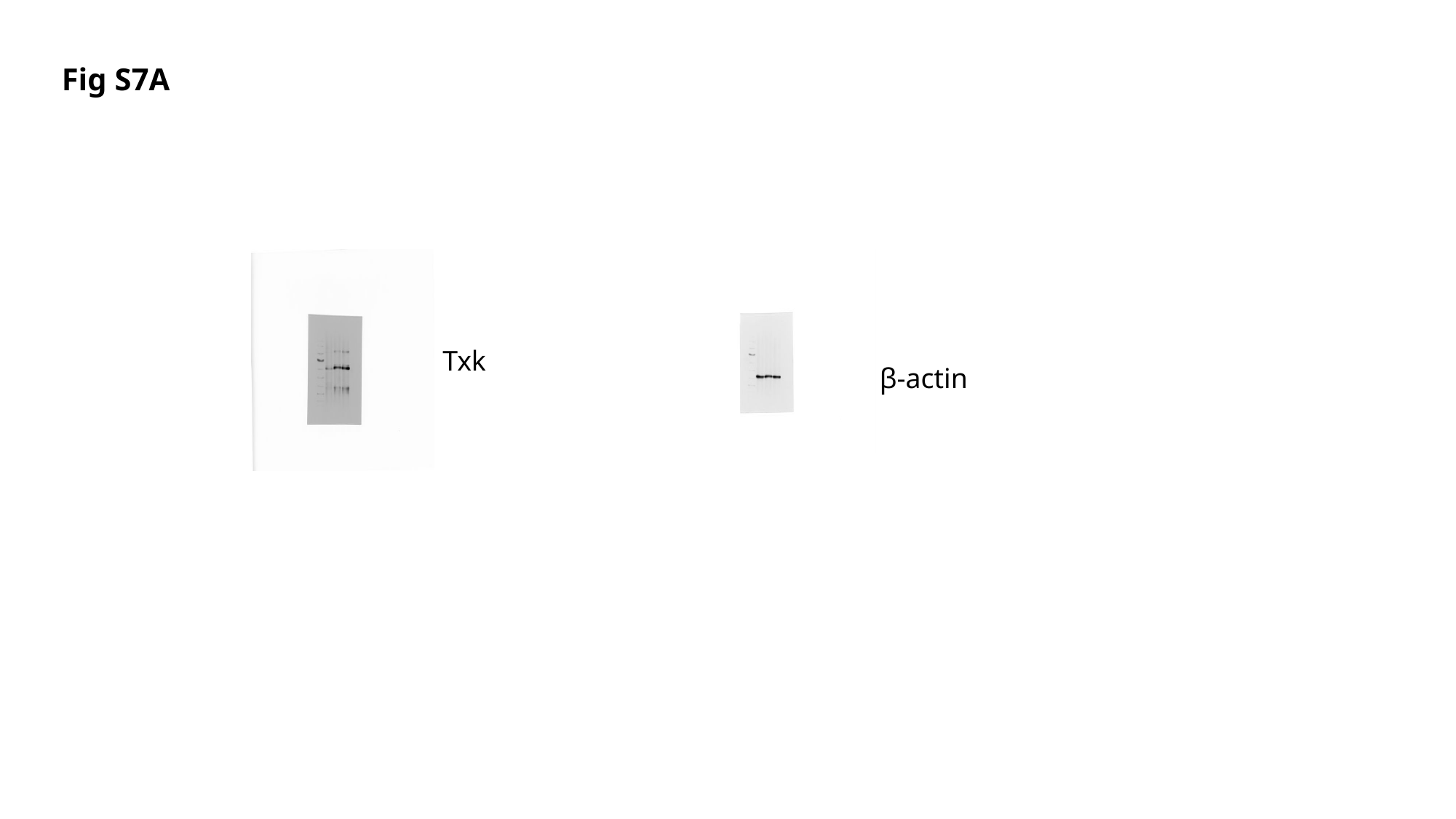

Fig S7A
Txk
β-actin

## Slide 6
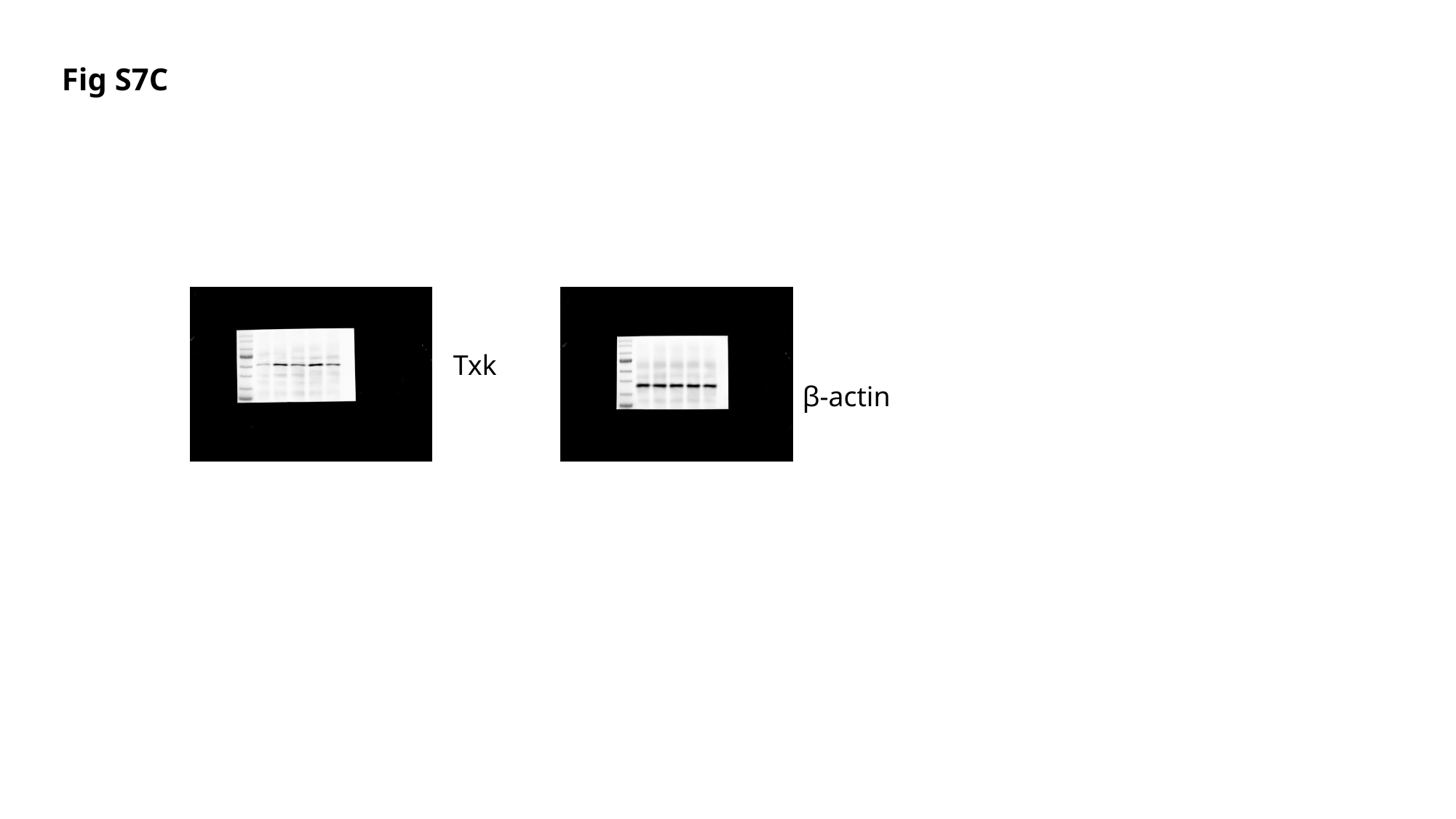

Fig S7C
Txk
β-actin
